# Immunoproteasomal Inhibition With ONX-0914 Attenuates Atherosclerosis and Reduces White Adipose Tissue Mass and Metabolic Syndrome in Mice

**DOI:** 10.1161/ATVBAHA.123.319701

**Published:** 2024-04-25

**Authors:** Frank H. Schaftenaar, Andrea D. van Dam, Gerjan de Bruin, Marie A.C. Depuydt, Jill de Mol, Jacob Amersfoort, Hidde Douna, Menno Meijer, Mara J. Kröner, Peter J. van Santbrink, Mireia N.A. Bernabé Kleijn, Gijs H.M. van Puijvelde, Bogdan I. Florea, Bram Slütter, Amanda C. Foks, Ilze Bot, Patrick C.N. Rensen, Johan Kuiper

**Affiliations:** 1 Division of BioTherapeutics, Leiden Academic Centre for Drug Research, the Netherlands (F.H.S., M.A.C.D., J.d.M., J.A., H.D., M.M., M.J.K., P.J.v.S., M.N.A.B.K., G.H.M.v.P., B.S., A.C.F., I.B., J.K.).; 2 Division of Endocrinology, Department of Medicine, Einthoven Laboratory for Experimental Vascular Medicine, Leiden University Medical Center, the Netherlands (A.D.D., P.C.N.R.).; 3 Department of Chemical Biology, Leiden Institute of Chemistry, the Netherlands (G.d.B., B.I.F.).

**Keywords:** atherosclerosis, dendritic cells, immunomodulation, inflammation, metabolic syndrome, proteasome inhibitors, T-lymphocytes

## Abstract

**BACKGROUND:**

Atherosclerosis is the major underlying pathology of cardiovascular disease and is driven by dyslipidemia and inflammation. Inhibition of the immunoproteasome, a proteasome variant that is predominantly expressed by immune cells and plays an important role in antigen presentation, has been shown to have immunosuppressive effects.

**METHODS:**

We assessed the effect of ONX-0914, an inhibitor of the immunoproteasomal catalytic subunits LMP7 (proteasome subunit β5i/large multifunctional peptidase 7) and LMP2 (proteasome subunit β1i/large multifunctional peptidase 2), on atherosclerosis and metabolism in LDLr^–/–^ and APOE*3-Leiden.CETP mice.

**RESULTS:**

ONX-0914 treatment significantly reduced atherosclerosis, reduced dendritic cell and macrophage levels and their activation, as well as the levels of antigen-experienced T cells during early plaque formation, and Th1 cells in advanced atherosclerosis in young and aged mice in various immune compartments. Additionally, ONX-0914 treatment led to a strong reduction in white adipose tissue mass and adipocyte progenitors, which coincided with neutrophil and macrophage accumulation in white adipose tissue. ONX-0914 reduced intestinal triglyceride uptake and gastric emptying, likely contributing to the reduction in white adipose tissue mass, as ONX-0914 did not increase energy expenditure or reduce total food intake. Concomitant with the reduction in white adipose tissue mass upon ONX-0914 treatment, we observed improvements in markers of metabolic syndrome, including lowered plasma triglyceride levels, insulin levels, and fasting blood glucose.

**CONCLUSIONS:**

We propose that immunoproteasomal inhibition reduces 3 major causes underlying cardiovascular disease, dyslipidemia, metabolic syndrome, and inflammation and is a new target in drug development for atherosclerosis treatment.

**Highlights**

- Immunoproteasome inhibition reduces atherosclerosis in LDLr^–/–^ mice.
- ONX-0914 treatment inhibits proatherogenic memory T cells, including Th1 cells, and antigen-presenting cells.
- Immunoproteasome inhibition depletes adipocyte progenitors and causes white adipose tissue inflammation.
- ONX-0914 reduces intestinal lipid uptake and gastric emptying.
- Immunoproteasomal inhibition reduces white adipose tissue mass and improves measures of metabolic syndrome.

Cardiovascular disease is the most common cause of death in the Western world with atherosclerosis as the main underlying pathology.^1^ Atherosclerosis is characterized by lipid deposition in the intima of medium- to large-sized arteries, which evokes immune infiltration in the arterial wall and inflammation. Current treatment of atherosclerosis primarily focuses on treating dyslipidemia in patients, which has led to a decrease in cardiovascular events but leaves substantial residual risk.^1^ The inflammatory response in atherosclerosis is characterized by a proinflammatory and pathogenic T cell response.^2^ The CANTOS trial (Canakinumab Anti-Inflammatory Thrombosis Outcome Study) provides evidence that immunomodulation, in addition to treating dyslipidemia, can lower the risk for major cardiovascular events.^3,4^

An emerging immune regulator is the immunoproteasome, a variant of the constitutive proteasome, which is expressed in all cell types. Immunoproteasomes are basally expressed in cells of hematopoietic origin but can also be induced in cells of nonhematopoietic origin through inflammatory stimuli, such as IFN (interferon)-γ.^5^ Proteasomes are responsible for the degradation of the vast majority of cellular proteins and involved in regulation of cellular processes via degradation of polyubiquitinated proteins.^6^

Structurally, proteasomes form a large barrel-like protein complex, the 20S proteasome, through axial stacking of 2 heptameric outer β-rings and 2 heptameric inner β-rings. Proteins are proteolytically cleaved within the proteasome by the lumen facing active sites of 3 distinct catalytically active β-subunits per β-ring. The catalytic subunits differ between the constitutive proteasome and immunoproteasome and have a slightly different substrate specificity.^7^ In the constitutive proteasome, the catalytic β1, β2, and β5 subunits exert the caspase-like, trypsin-like, and chymotrypsin-like activity, whereas β1i (LMP2 [proteasome subunit β1i/large multifunctional peptidase 2]), β2i (MECL-1), and β5i (LMP7 [proteasome subunit β5i/large multifunctional peptidase 7]) subunits exert the proteolytic activity in the immunoproteasome.^8^

Immunoproteasomes strongly support CD8^+^ T cell responses since they are more effective at generating MHC-I epitopes compared with constitutive proteasomes.^7^ Using the small-molecule ONX-0914 (formerly known as PR-957), an inhibitor of the immunoproteasome subunits LMP7 and LMP2 (LMP7/2), it was demonstrated that, besides CD8^+^ T cell activation, the immunoproteasome is also involved in the regulation of multiple other inflammatory processes.^9,10^ For example, ONX-0914 reduces inflammatory cytokine secretion by peripheral blood mononuclear cells and T cells.^9,11^ In addition, ONX-0914 skews T cell differentiation, which limits CD4^+^ T cell differentiation toward the proinflammatory Th1 and Th17 helper T cells and promotes differentiation toward Tregs.^12^ LMP7/2 inhibition also affects dendritic cell (DC) functionality by inhibiting the capacity of DCs to prime T cells.^13^ In various murine models of autoimmune diseases, such as experimental autoimmune encephalomyelitis and rheumatoid arthritis, the immune inhibitory effects of immunoproteasomal inhibition with ONX-0914 reduce disease severity.^9–12,14–18^

Recently, it was shown that immunoproteasomal inhibition can also reduce early atherosclerosis, concurrent with an increase in efferocytosis of apoptotic cells in the plaque.^19^ We further characterized the effects of immunoproteasomal inhibition in an atherosclerotic setting and show that ONX-0914 treatment reduces macrophage, DC, and T cell activation. We also found that immunoproteasomal inhibition has marked effects on metabolic syndrome, reducing intestinal lipid uptake and gastric emptying, and reducing white adipose tissue mass. Our data indicate that immunoproteasomal inhibition can form a highly useful approach to address atherosclerosis and metabolic syndrome.

## MATERIALS AND METHODS

The data that support the findings of this study are available from the corresponding author upon reasonable request.

### Animals

All animal work was approved by the Leiden University Animal Ethics Committee, and the animal experiments were performed conform to the guidelines from Directive 2010/63/EU of the European Parliament on the protection of animals used for scientific purposes. Mice were housed in individual ventilated cages with aspen bedding, in groups of 2 to 4 mice per cage except for the duration of the metabolic cage measurements, during which mice were single-housed. Mice were fed a standard laboratory diet (Special Diets Services, RM3[P], 4.16% crude oil, 21.86% crude protein, 33.61% starch, and 5.84% sugar) before initiation of the in vivo experiments after which LDLr^–/–^ (C57BL/6J background, Jackson Laboratory; original purchase, further bred in house) were fed a Western-type diet (WTD) containing 0.25% cholesterol, 15% cocoa butter (Special Diet Services, Witham, Essex, United Kingdom), and C57BL/6J APOE*3-Leiden.CETP (E3L.CETP) mice^20^ (C57BL/6J background) were fed a WTD containing 0.1% cholesterol and 15% cocoa butter (HopeFarms; Woerden, the Netherlands) for indicated durations.

Mice were intraperitoneally treated with ONX-0914 (synthesized by the Leiden Institute of Chemistry) at a concentration of 10 mg/kg, 100 to 150 µL per injection, 3× weekly for the indicated durations. ONX-0914 was solubilized in DMSO after which it was diluted in PBS (37 °C) to prevent precipitation of ONX-0914 (4% DMSO). Four percent of DMSO in PBS served as a control treatment. At the end point of the studies using LDLr^–/–^ mice, mice were anesthetized by subcutaneous injection with a mix of ketamine (100 mg/mL), sedazine (25 mg/mL), and atropine (0.5 mg/mL) and retro-orbitally exsanguinated, and perfused with PBS. Directly after the final blood withdrawal in E3L.CETP mice, mice were euthanized by cervical dislocation, and perfused with PBS to stop the uptake of lipids from VLDL (very low-density lipoprotein)-like particles.

To investigate gastric emptying, GastroSense 750 (Perkin Elmer) was administered by oral gavage, according to manufacturer’s recommendations, and mice were euthanized 30 minutes later by cervical dislocation. The gastrointestinal tract was excised and imaged using the IVIS Spectrum in vivo imaging system. The average luminescence in the stomach was determined using ImageJ. The reported numbers in the figure legends refer to biological replicates.

### Insulin Tolerance Test and Insulin Tolerance Test

The insulin tolerance test (ITT) and glucose tolerance test (GTT) were performed in male LDLr^–/–^ mice (n=7 per group; GTT, 62 days; ITT, 77 days of age). To develop obesity and metabolic syndrome, the mice were fed a WTD for 7 weeks. Thereafter, mice remained on a WTD and were treated with ONX-0914 (10 mg/kg, 3× weekly) or control. At 8:30 (start of light phase), mice were fasted for 6 hours before the ITT or GTT. After 6 hours of fasting, a baseline blood sample was taken from the lateral tail vein to determine blood glucose levels (measured with Accu-Chek instant system). For the glucose tolerance test, 0.06 g glucose (Sigma) was orally administered (dose of 2 g/kg for a 30-g mouse). Thereafter, blood glucose levels were measured 15, 30, 60, 90, and 120 minutes after glucose administration. For the ITT, mice received 0.03 units of insulin (Roche) intraperitoneally (1 U/kg for a 30-g mouse). Then, blood glucose levels were measured 15, 30, 60, and 90 minutes after insulin administration. When blood glucose levels dipped below 2 mmol/L mice were humanely euthanized.

### Histology

Hearts were transversally cut in half and incubated in OCT medium for 30 minutes. After 30 minutes, hearts were fast frozen on dry ice and stored at −80 °C before cryosections (10 µm) of the aortic root were collected on Superfrost Plus Adhesion Microscope Slides (Thermo Fisher Scientific) and analyzed at 70-µm intervals (7 slides/mice). Neutral fats were stained with oil red O in the last 5 sections of the aortic root containing valvular leaflets, to determine the average atherosclerotic lesion size in the aortic root. Lesion collagen content was determined with Masson trichrome staining (Sigma-Aldrich) for 3 subsequent sections (corresponding to sections 2 to 4 of the oil red O staining). Corresponding sections were immunohistochemically stained for macrophages with MOMA-2 (monocyte-macrophage-2) antibody (Sanbio, 1:1000 dilution). Slides were blocked with 5% milk powder before primary antibody was added for 2 hours at RT, after which primary antibody was incubated overnight at 4 °C. Then slides were incubated with Goat anti-rat Ig alkaline phosphatase (A8438, Sigma-Aldrich) for 1 hour at RT, after which BCIP (5-bromo-4-chloro-3-indolyl phosphate)/NBT (nitro blue tetrazolium) substrate (DAKO) was used to stain macrophages blue. For fluorescent staining of CD68 (FA-11, BioLegend, 1:800 dilution), α-SMA (1A4, Sigma-Aldrich, 1:1000 dilution) samples were blocked with 5% milk powder and incubated overnight with the preconjugated antibodies at 4 °C after which tissue sections were washed and embedded with fluoroshield-containing DAPI (4′,6-diamidino-2-phenylindole; Sigma-Aldrich). Blinded histological analysis was performed using a Leica DM-RE microscope, a Pannoramic 250 Flash slidescanner (3dHISTECH), LeicaQwin software (Leica Imaging Systems, Cambridge, United Kingdom), ImageJ, and QuPath.

### gWAT Digestion, SVF and Primary Adipocyte Isolation, and Primary Adipocyte Culture

After perfusion with PBS, perigonadal white adipose tissue (gWAT) was excised and minced in DPBS (Lonza) supplemented with 0.5% BSA (Sigma). Thereafter, minced adipose tissue was incubated in DPBS with 10 mmol/L CaCl_2_, and 4 mg/mL collagenase type II derived from *Clostridium histolyticum* (Sigma) at 37 °C, while gently agitated in a rotational shaker. Adipose tissue homogenates were passed over a 300-µm mesh (Elko, Fisher Scientific) and thereafter centrifuged at 150*g* for 10 minutes at room temperature. Infranatant was removed, saved for assessment of stromal vascular fraction (SVF) cell populations with flow cytometry or discarded, and remaining floating adipocytes were washed twice with DPBS (Gibco) supplemented with 0.5% BSA. Adipocytes were either directly taken up in GTC (guanidine thiocyanate) and stored at −80 °C for gene expression analysis, or cultured overnight in DMEM (Dulbecco’s Modified Eagle Medium; Lonza) supplemented with 10% FCS (GE Healthcare Life Sciences), 100 U/mL penicillin/streptomycin (GE Healthcare Life Sciences), and 2-mM l-glutamine (Thermo Fisher Scientific).

### Flow Cytometry

Extracellular staining of single-cell suspensions was performed in PBS with 2% FCS and αCD16/32 antibody (93, BioLegend) and eBioscience Fixable Viability Dye eFluor 780 (Thermo Fisher Scientific) to discriminate between living and dead cells for 30 minutes at 4 °C. For intracellular transcription factor staining after extracellular staining, cells were fixed and permeabilized with the FoxP3 transcription factor buffer set (Thermo Fisher Scientific/eBioscience) according to manufacturer’s instructions, and incubated with flow cytometry antibodies for 45 minutes at 4 °C. Spleen and lymph nodes were mashed over a 70-µm cell strainer (Greiner) to obtain single-cell suspensions and red blood cells were lysed with ACK (ammonium-chloride-potassium) lysis buffer if necessary. A list of antibodies used during this study is present in the Major Resources Table in the Supplemental Material.

Compensation measurements were performed using UltraComp eBeads (Thermo Fisher Scientific) and ArC Amine-Reactive Compensation Beads (Thermo Fisher Scientific). Cells were measured with a FACSCanto II (BD Biosciences) and a Cytoflex S flow cytometer (Beckman Coulter) and analyzed using FlowJo software (Tree Star, Inc).

### Thymidine Incorporation Assay

Splenocytes (2×10^5^ cells/well) were stimulated with anti-CD3e (1 μg/mL) and anti-CD28 (0.5 μg/mL; both from Thermo Fisher Scientific) for 72 hours and incubated with 0.5 μCi/well ^3^H-thymidine (Perkin Elmer) for the last 16 hours, or remained unstimulated. Cells were thoroughly washed with PBS and thereafter lysed with natriumhydroxide and taken up in Emulsifier-Safe (Perkin Elmer). ^3^H-thymidine incorporation was measured using a liquid scintillation analyzer (Tri-Carb 2900R). Responses are expressed as the mean disintegrations per minute. The stimulation index was defined by dividing the disintegrations per minute under activated conditions by the disintegrations per minute under nonactivated conditions per mouse.

### Indirect Calorimetry

After 1 day of acclimatization, O_2_ consumption, CO_2_ production, and food intake were measured for 6 consecutive days in fully automatic metabolic cages (LabMaster System, TSE Systems, Bad Homburg, Germany). Total energy expenditure was estimated from the VO_2_ and resting energy requirement. Carbohydrate oxidation was calculated using the formula ([4.585×VCO_2_]−[3.226×VO_2_])×4, in which the 4 represents the conversion from mass per time unit to kilo calories per time unit.^21^ Similarly, fat oxidation was calculated using the formula ([1.695×VO_2_]−[1.701×VCO])×9. Physical activity was monitored using infrared sensor frames. The first 24 hours directly after injection with ONX-0914 or vehicle (day of treatment) were analyzed and graphed separately from the same 24-hour period 24 hours later (day after treatment) and subdivided into light phase and dark phase.

### Preparation of VLDL-Like Triglyceride-Rich Emulsion Particles and Clearance Assay

VLDL-like triglyceride-rich emulsion particles (80 nm) containing radiolabeled glycerol tri[^3^H]oleate and [^14^C] cholesteryl oleate were synthesized like previously.^22^ In brief, emulsion particles were obtained by sonicating a mixture of trioleate (70 mg), egg yolk phosphatidylcholine (22.7 mg), cholesteryl oleate (3.0 mg), lysophosphatidylcholine (2.3 mg), and cholesterol (2.0 mg), containing tri[^3^H]oleate (100 μCi) and [^14^C] cholesteryl oleate (10 μCi) tracers, at 54 °C using a Soniprep 150 (MSE Scientific Instruments, United Kingdom) set at 10 µm output. VLDL-like particles were obtained through density gradient ultracentrifugation using a Beckman SW 40 Ti rotor. First chylomicron-like particles were discarded by removing the top fraction after centrifugation (20 000 rpm, 27 minutes, 20 °C); thereafter, the remainder was centrifuged (40 000 rpm, 27 minutes, 20 °C) and VLDL-like particles were isolated and stored at 4 °C under argon and used within 5 days.

To study the in vivo clearance of cholesterol and triglyceride, E3L.CETP mice treated for 2 weeks received a final ONX-0914 treatment and, directly after that, were fasted for 4 hours after which a baseline blood sample was drawn, and VLDL-like emulsion particles were IV administered. Two, 5, 10, and 15 minutes after administration of the emulsion particles, blood samples were drawn. Blood samples were obtained by tail bleeding using paraoxon (Sigma)-coated heparinized capillary tubes (Hawksley, Sussex, England). [^3^H] and [^14^C] radioactivity was determined in plasma. Total plasma volume was estimated by multiplying body weight (g) with 0.04706, as previously described.^23^ Half-life times were derived from the half-life constant, which was calculated using the log values for [^3^H] and [^14^C] measurements for *t*=2, 5, and 10 minutes. After the final blood withdrawal, mice were euthanized by cervical dislocation and perfused with PBS after which organs were collected and weighed for determination of [^3^H] and [^14^C], and gene expression.

### Oral Triglyceride Loading Test and Plasma Lipoprotein Analysis

Mice were fasted overnight, after which a baseline blood sample was drawn. Mice received an oral bolus of 200-µL olive oil, and blood was drawn 30, 60, 120, 180, and 240 minutes after that. Blood was collected in Microvette CB300 lithium-heparin-coated capillary tubes (Sarstedt).

Triglyceride and total cholesterol levels in blood plasma from the oral triglyceride loading test and other experiments were assessed with commercially available kits (Roche). HDL (high-density lipoprotein) cholesterol levels were measured in supernatant from blood plasma, after ApoB-containing lipoproteins were precipitated by the addition of 20% polyethylene glycol in 200-mmol/L glycine buffer (pH, 10). The activity of aspartate aminotransferase and alanine aminotransferase in blood plasma was assessed with activity assay kits (Sigma, MAK055, and MAK052, respectively) according to the manufacturer’s protocols.

### Real-Time Quantitative PCR

RNA was extracted from adipocytes, and mechanically disrupted gWAT, interscapular brown adipose tissue, liver, and spleen, using Trizol reagent following manufacturer’s instructions (Invitrogen). Thereafter, cDNA was generated using RevertAid M-MuLV reverse transcriptase according to manufacturer’s protocol (Thermo Fisher Scientific). Quantitative gene expression was measured using Power SYBR Green Master Mix (Thermo Fisher Scientific) on a 7500 Fast Real-Time PCR system (Applied Biosystems). Gene expression was normalized to housekeeping genes Actb and Rplp0. A list of the primers can be found in Table S1.

### Multiplex ELISA

Inflammatory cytokines in blood plasma and culture supernatant of splenocytes stimulated overnight with anti-CD3e (1 μg/mL) and anti-CD28 (0.5 μg/mL) were assessed with a T cell differentiation 17-plex Luminex bead-based assay (eBioscience, EPX170-26087-901) according to manufacturer’s instructions. Metabolic syndrome–related biomarkers were assessed in blood plasma with a diabetes 8-plex Luminex bead-based assay (Bio-Rad, 171F7001M) following the manufacturer’s protocol. Luminex assays were measured on a MAGPIX System (Luminex).

### Western Blot

A quarter of spleen tissue was pottered and lysed in 500-µL lysis buffer, consisting of 40-mmol/L KCl, 50-mmol/L Tris-base, 5-mmol/L MgCl_2_, and 1-mM DTT (freshly added). Protein content was determined with a BCA assay (Pierce, Thermo Fisher Scientific). Protein lysates were incubated in Laemmli sample buffer at 95 °C for 10 minutes. Ten micrograms of protein lysates were run on 12.5% acrylamide gels with a 29:1 ratio of acryl:bis-acry (Bio-Rad: catalog No. 161-0156) in SDS-electrophoresis buffer. PageRuler Plus Prestained Protein Ladder (10–250 kDa, Thermo Fisher Scientific) was used as standard. Gel electrophoresis was performed for 30 minutes at 60 V, then 120 V until the blue color reached the bottom of the gel in the Mini-PROTEAN Tetra Cell system (Bio-rad). Proteins were wet-blotted on a PVDF (polyvinylidene fluoride) membrane (Whatman) with a blot buffer (pH, 8.3) consisting of 25 mmol/L Tris-base, 190 mmol/L glycine, and 20% methanol on ice at 200 mA for 30 minutes in a Mini Trans-Blot Cell (Bio-Rad). Blots were dried for 45 minutes to enhance protein binding to the PVDF membrane. Blots were (re-)activated with 100% methanol (≈20 seconds), washed in TBS-T, and blocked with 5% BSA in TBS-T for 1 hour. Blots were incubated with a 1:1000 dilution of polyclonal rabbit antibody against LMP7 antibody (Abcam, ab3329), β5 (Abcam, ab3330), or β-actin (Abcam, ab8227) in TBS-T with 1% BSA overnight at 4 °C with constant agitation. After washing in TBS-T, blots were incubated with a 1:2500 dilution of goat anti-rabbit antibody conjugated with HRP (Abcam, ab205718) in TBS-T with 1% BSA for 1 hour at room temperature. Western blots were visualized using SuperSignal West Pico Chemiluminescent Substrate (Thermo Fisher Scientific) with an Amersham Imager 600.

### Statistics

To identify statistical outliers, we used the robust regression and outlier removal method with a quality Q of 1. Comparative analyses between 2 groups were performed using Student *t* test, although 1- or 2-way ANOVA followed by Šídák post hoc tests were utilized for multigroup comparisons. A significance threshold of *P*<0.05 was adopted. All statistical computations were executed using GraphPad Prism 9.0.0 (GraphPad).

## RESULTS

### ONX-0914 Treatment Reduces Atherosclerosis

Since pharmacological inhibition of the immunoproteasomal active subunits LMP7/2 reduced inflammation and disease severity in experimental models of autoimmune diseases,^9–12,14–18^ we aimed to assess the effect of LMP7/2 inhibition on atherosclerosis. ONX-0914 was previously reported to specifically inhibit the immunoproteasome at a dose of 10 mg/kg.^9^ We assessed whether this dose also specifically inhibited the immunoproteasome in hyperlipidemic mice by Western blotting for LMP7, and the constitutive proteasome subunit β5 in spleen lysates from LDLr^–/–^ mice fed a WTD for 27 weeks and treated with ONX-0914 (10 mg/kg) or control for 1 week. ONX-0914 covalently bound and inactivated 73.4±8.9% of LMP7 (Figure S1A). From all constitutive catalytic proteasome subunits, ONX-0914 binds the constitutive β5 subunit with the highest affinity.^9^ The β5 subunit remained uninhibited and was upregulated by 70.5±46.4% by ONX-0914 treatment compared with control (Figure S1A).^20–22^ These data demonstrate that a dose of 10-mg/kg ONX-0914 specifically and effectively inhibits the LMP7 subunit of the immunoproteasome in hyperlipidemic mice.

**Figure 1.**
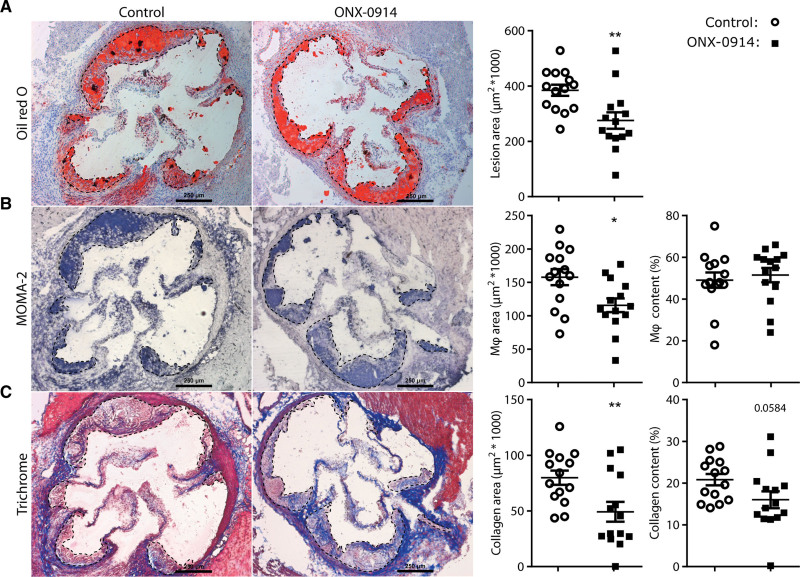
**ONX-0914 treatment reduces atherosclerosis in aortic root.** Female LDLr^–/–^ (n=15) were treated with ONX-0914 (10 mg/kg, 3× weekly intraperitoneally) or control-treated for 7 weeks, while fed a Western-type diet (WTD). Thereafter, mice were euthanized and (**A**) lipids in the aortic root were stained with oil red O to quantify the atherosclerotic lesion area. **B**, Aortic root sections were immunohistochemically stained using MOMA-2 (monocyte-macrophage-2) antibody to determine macrophage area and macrophage content of the plaque. **C**, Masson Trichrome staining was used to assess collagen area and collagen content of the plaque. Expressed as mean±SEM, *P<0.05, ** *P*<0.01 (**A–C**: Student *t* tests), scale bars are 250 µm.

To address its therapeutic potential for the treatment of atherosclerosis, LDLr^–/–^ mice were treated with ONX-0914 (10 mg/kg) 3× weekly for 7 weeks while fed a WTD. Analysis of the atherosclerotic lesions in the aortic root (Figure 1A) revealed a 28.4% reduction (*P*<0.01) in lesion size in the ONX-0914–treated group (2.8±0.5×10^5^ µm^2^) compared with the vehicle-treated group (3.8±0.7×10^5^ µm^2^). The absolute MOMA-2^+^ macrophage area in the lesions was reduced by 26.6% upon LMP7/2 inhibition, while the relative macrophage area in the plaque was not affected (Figure 1B). The absolute collagen area in the plaque was reduced (−38.3%), and a clear trend toward a lower relative collagen content of the plaque (−23%; *P*=0.058) was demonstrated in the ONX-0914–treated group (Figure 1C), indicating a less progressed plaque phenotype upon ONX-0914 treatment.

### ONX-0914 Reduces DC and Macrophage Levels and Activation

**Figure 2.**
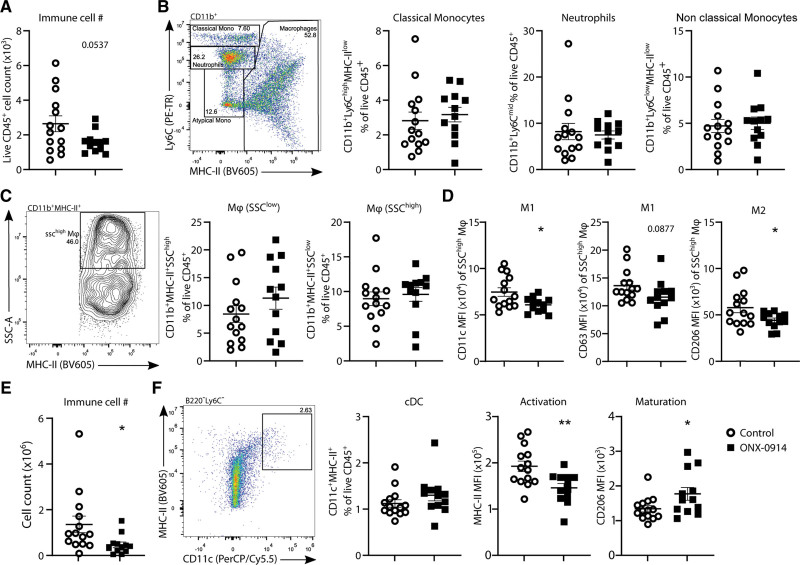
**Effect of ONX-0914 on myeloid cell populations in the aortic arch and HLN.** Flow cytometric analysis of myeloid cells in (**A–D**) aortic arch and (**E** and **F**) HLN of aged (68–73 weeks old) male LDLr^–/–^ mice (n=14-13), treated with ONX-0914 (10 mg/kg, 3× weekly intraperitoneally) or control for 6 weeks while remaining on a standard laboratory diet. **A**, Total immune cell number in the aortic arch. **B**, Gating of various myeloid populations in a concatenated sample containing live CD45^+^CD11b^+^ from all mice and quantification per mouse. **C**, Gating of SSC^low^ and SSC^high^ macrophage populations in a concatenated sample containing live CD45^+^CD11b^+^MHC-II^+^ from all mice and quantification per mouse. **D**, Quantifications of M1 and M2 marker expression in the SSC^high^ macrophage subset. **E**, Total immune cell number in the HLN. **F**, Gating of conventional dendritic cells (cDCs) from live B220^−^Ly6C^−^ cells in a representative HLN sample, and quantification of cDC levels and activation and maturation. Expressed as mean±SEM, unpaired *t* test, *P<0.05, **P<0.01 (**A–F**: Student *t* tests).

To assess the effect of immunoproteasomal inhibition on plaque immune cells, we assessed the immune cells in the aortic arch of standard laboratory diet fed aged (68–73 weeks of age) male LDLr^–/–^ mice, treated with ONX-0914 or control-treated for 6 weeks. In this atherosclerosis model, mice gradually develop collagen-rich plaques while the aging immune system also adopts a more proinflammatory phenotype, as part of a process often referred to as inflammaging.^24^ The plaques in this model tend to be pretty advanced and stable, which allows to study the effect of an intervention on the immune system with little bias from a change in the developmental stage of the plaque. In line with this more stable plaque phenotype, we did not observe an effect of ONX-0914 treatment on plaque size (Figure S2A), plaque collagen content (Figure S2B), plaque necrotic core area (Figure S2B), plaque macrophage content (Figure S2C), and plaque smooth muscle cell content (Figure S2C) in the aortic root. With flow cytometry, we further assessed the immune cells in the plaque-bearing aortic arch. We found a trend toward a reduction in overall immune cell number (*P*=0.054) in the ONX-0914–treated group (Figure 2A). However, in percentage the levels of classical monocytes (Figure 2B), nonclassical monocytes (Figure 2B), neutrophils (Figure 2B), macrophages (Figure 2C), B cells (data not shown), and T cells (data not shown), remained stable, indicating that not a single particular immune population was responsible for the observed trend. The SSC^high^ macrophage population in the aortic arch did have a more immature phenotype in the ONX-0914–treated group, with a lower expression of CD11c (M1), a trend toward a lower CD63 expression (M1; *P*=0.088), and a reduction of CD206 (M2) expression (Figure 2D). As we observed a less mature phenotype of plaque macrophages, we assessed whether ONX-0914 treatment also impacted antigen-presenting cells in lymph nodes draining from the heart and aortic arch. ONX-0914 reduced the total immune cell number in the mediastinal lymph nodes (Figure 2E), indicative of reduced vascular inflammation. DC levels were constant in percentage between treatment groups; however, the DCs had a less activated and more immature phenotype in the ONX-0914–treated group (Figure 2F).

**Figure 3.**
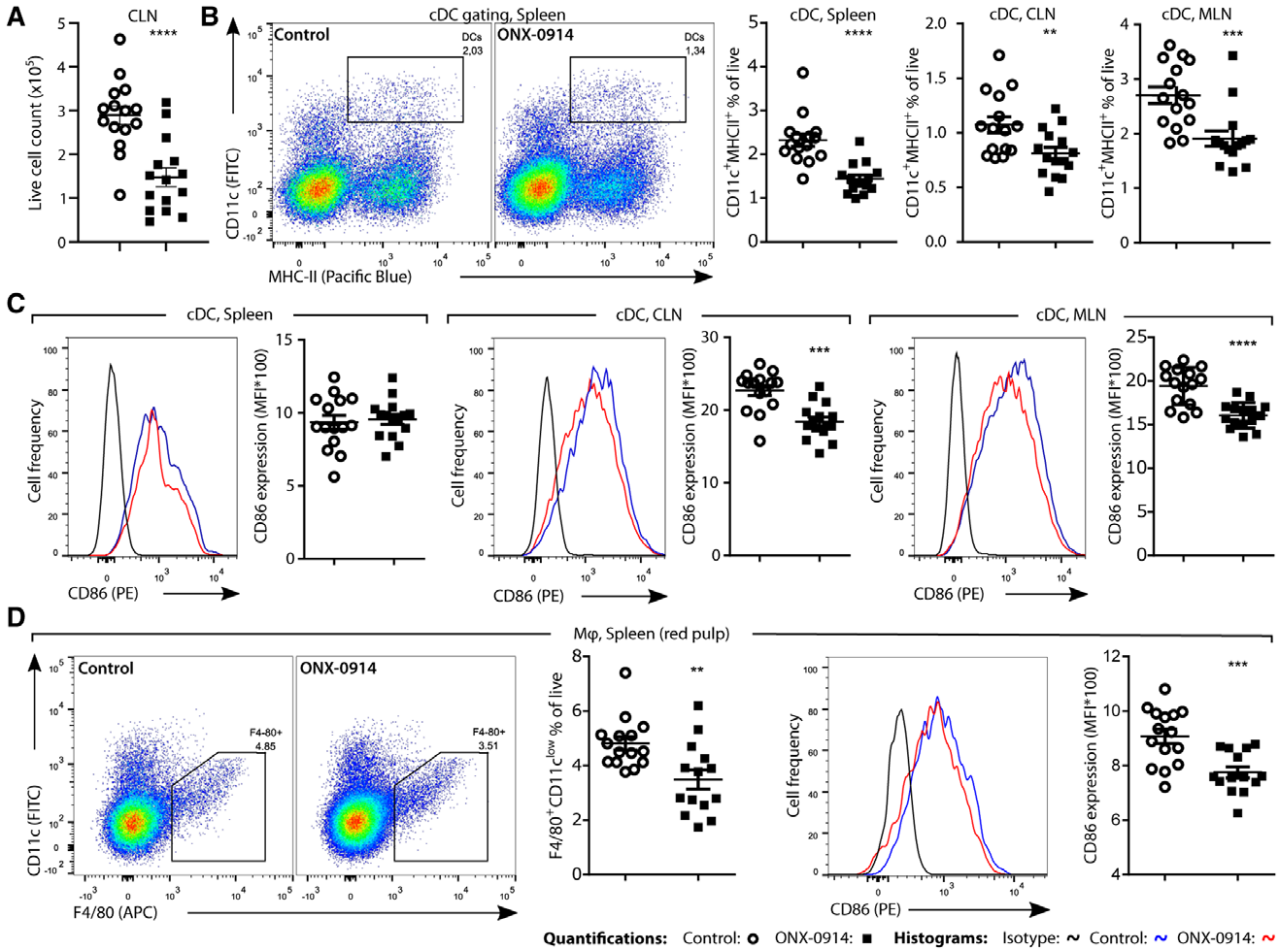
**ONX-0914 lowers conventional dendritic cell (DC) and red pulp macrophage levels and activation.** Female LDLr^–/–^ (n=15) were control-treated or treated with ONX-0914 (10 mg/kg, 3× weekly intraperitoneally) for 7 weeks. **A**, The cell number in the CLN counted by flow cytometry. **B**, Representative flow cytometry plots from spleen, depicting gating of cDCs in the living singlets gate, based on expression of CD11c and MHC-II, quantified in spleen, CLN, and MLN. **C**, Representative histograms of expression and quantification of median fluorescent intensity (MFI) of CD86 on cDCs in the spleen, CLN, and MLN. **D**, Representative flow cytometry plots from the spleen depicting gating of F4/80^+^CD11c^low^ red pulp macrophages from living singlets minus cDCs quantified in the spleen and a representative histogram of CD86 expression on splenic red pulp macrophages and quantification. Expressed as mean±SEM, **P<0.01, ***P<0.001, ****P<0.0001 (**A–D**: Student *t* tests).

We assessed whether there was a similar effect of ONX-0914 on overall immune cell levels, and DCs and macrophages in several lymphoid organs in the atherosclerosis initiation study. The aortic arch draining cervical lymph nodes (CLNs) contained fewer immune cells in the ONX-0914–treated group (Figure 3A), although mesenteric lymph node (MLN) cell count and spleen weight were similar between the treatment groups (data not shown). ONX-0914 treatment significantly reduced the levels of conventional CD11c^+^MHC-II^+^ DCs in the spleen, CLN draining the aorta,^25^ and MLN (Figure 3B). Activation of conventional DCs was slightly lowered in the CLNs and MLNs upon LMP7/2 inhibition, as judged by the lower expression of CD86 (Figure 3C). Splenic DCs did not exhibit a decrease in CD86 expression due to ONX-0914 treatment, possibly due to a less activated DC population present in the spleen compared with the lymph nodes, reflected by the lower CD86 expression in splenic DCs compared with DCs derived from lymph nodes of control-treated mice (Figure 3C). Furthermore, ONX-0914 reduced F4/80^+^ red pulp macrophage levels and their activation in the spleen (Figure 3D). To establish whether the reduction in DC levels and activation were direct of the effect of ONX-0914 and not a result of a reduction in atherosclerosis, we performed a bone marrow–derived DC culture. We found that ONX-0914 reduced bone marrow–derived DC viability in a concentration-dependent manner (Figure S3A through S3C), and a trend toward reduced CD86 expression (Figure S3B), similar to the in vivo observations. Furthermore, we observed increased expression of constitutive catalytic proteasome subunits in the bone marrow–derived DC upon ONX-0914 treatment (Figure S3D), providing further evidence for compensatory upregulation of constitutive proteasome subunits upon immunoproteasomal inhibition. In contrast to the constitutive proteasome catalytic subunit expression, expression of immunoproteasome catalytic subunits and immunoproteasome regulator subunits proteasome activator (PA)28α and PA28β was reduced in BMDCs upon ONX-0914 treatment (Figure S3C), likely impacting which proteins are degraded in the cell.

Since proinflammatory DCs^26^ and macrophages^27,28^ promote atherogenesis, a reduction in DC and macrophage levels and activation upon ONX-0914 treatment are likely to have contributed to the observed reduction in atherosclerosis.

### ONX-0914 Treatment Reduces Memory T Cell Levels

Because atherosclerosis is marked by a pathogenic CD4^+^ T cell response,^2^ and we observed reduced number and activation of APCs, we assessed whether ONX-0914 treatment skewed T helper differentiation away from Th1 cells and toward atheroprotective Tregs, in line with previously described in vitro and in vivo data.^12^ We did however not observe ONX-0914–mediated induction of CD4^+^ Tregs by flow cytometry in the spleen, blood, CLN, and MLN (Figure S4A).

Splenic T cell proliferative capacity and T cell differentiation were unaltered upon ONX-0914 treatment in this early atherosclerotic setting, as judged by similar thymidine incorporation in splenocytes (Figure S5A), and IFN-γ, IL (interleukin)-4, IL-17a, and IL-10 levels in culture supernatant of αCD3/αCD28-stimulated splenocytes (Figure S5B). Further, in line with these data, we did not observe changes in CD4^+^ (Figure S5C) or CD8^+^ T cell (Figure S5E) levels, and central memory T cell (Tcm), effector memory T cell (Tem), and naive (Th0/Tc0) T cell levels after ONX-0914 treatment in the spleen (Figure S5D through S5F).

**Figure 4.**
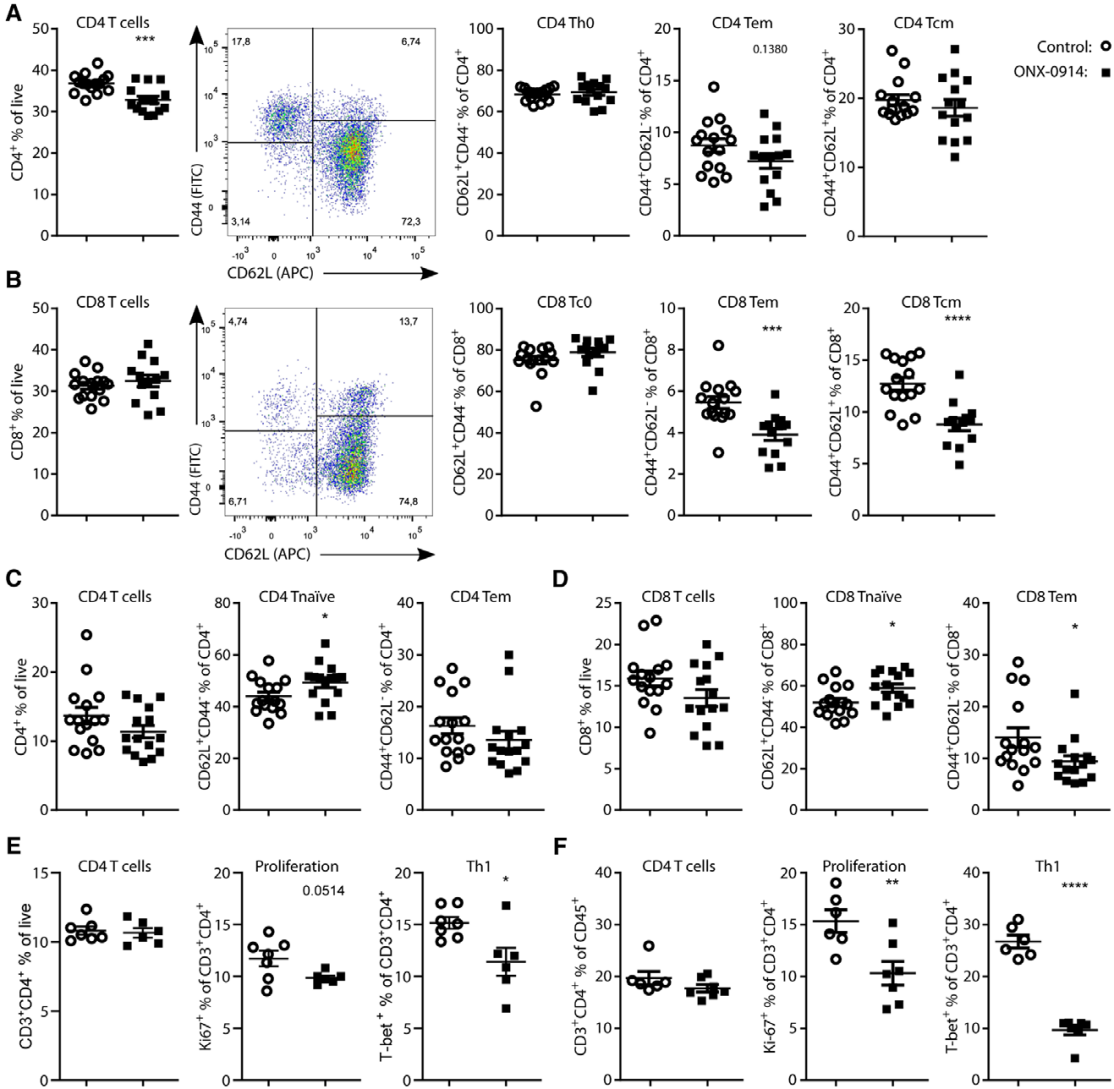
**ONX-0914 treatment attenuates maturation of T cells in vivo.** Flow cytometric analysis of naive (CD62L^+^CD44^−^), effector memory T cell (Tem; CD44^+^CD62L^−^), and Tcm (CD44^+^CD62L^+^), CD4^+^ T cells and CD8^+^ T cells in the mesenteric lymph node (**A** and **B**), and in the blood (**C** and **D**) of female LDLr^–/–^ mice (n=15) treated with ONX-0914 (10 mg/kg, 3× weekly intraperitoneally) or control-treated for 7 weeks although on a Western-type diet (WTD). **E** and **F**, Quantifications of flow cytometric analysis of CD4^+^ T cell levels, CD4^+^ T cell proliferation, and Th1 cell levels in spleens from animals derived from 2 experimental setups of advanced atherosclerosis. **E**, Aged male LDLr^–/–^ mice (68–73 weeks of age before WTD feeding) fed a WTD for 6 weeks before sacrifice, during which mice were treated with ONX-0914 (10 mg/kg, 3× weekly intraperitoneally) or control. **F**, Female LDLr^–/–^ mice (n=7) were fed a WTD for 27 weeks and treated with ONX-0914 (10 mg/kg, 3× weekly intraperitoneally) or control in the final week of WTD before sacrifice. Expressed as mean±SEM, *P<0.05, **P<0.01, ***P<0.001, ****P<0.0001 (**A–F**: Student *t* tests).

Despite the overall reduction in immune cells in the CLN, we did not observe an effect on the memory T cell population levels in the CLN of ONX-0914–treated mice (Figure S6A through S6D). ONX-0914 treatment did cause an overall reduction of CD4^+^ T cell levels and a trend (*P*=0.14) toward a reduction in CD4^+^ Tem cells in the MLN (Figure 4A). Furthermore, LMP7/2 inhibition reduced CD8^+^ Tem and Tcm cells in the MLN, while overall CD8^+^ T cell levels were unchanged (Figure 4C) and slightly increased in percentage in the CLN (Figure S6C). In the blood, overall CD4^+^ and CD8^+^ T cell levels were not significantly altered but naive CD4^+^ T cell (Figure 4C) and naive CD8^+^ T cell levels were increased (Figure 4D), coinciding with lowered CD8^+^ Tem cells (Figure 4D). Thus, although ONX-0914 treatment does reduce Tem and Tcm cells, the effects were modest and only significant in the secondary lymphoid organs that were analyzed.

A potential explanation for the modest effects on Tem and Tcm cells could be that in this experimental set up for atherosclerosis initiation (LDLr^–/–^ on WTD for 7 weeks), the mice had a relatively large population of naive T cells. We, therefore, assessed whether immunoproteasomal inhibition could reduce Th1 cells in more advanced stages of atherosclerosis using 2 different experimental approaches. In the first set up, male LDLr^–/–^ mice were aged on a standard laboratory diet for 68 to 73 weeks upon which mice were treated with ONX-0914 or control for 6 weeks while on a WTD. In the second set up, 10- to 14-week-old mice were fed a WTD for 27 weeks and treated with ONX-0914 in the last week. In both experimental setups, ONX-0914 reduced Th1 levels and CD4^+^ T cell proliferation (Figure 4E and 4F) but did not increase regulatory CD4^+^ T cell levels (Figure S4B and S4C).

These data indicate that immunoproteasomal inhibition has the potential to inhibit the pathogenic T cell response in atherosclerosis but does not appear to induce regulatory T cells.

### ONX-0914 Reduces Markers of Metabolic Syndrome While Inducing Signs of Innate Inflammation

**Figure 5.**
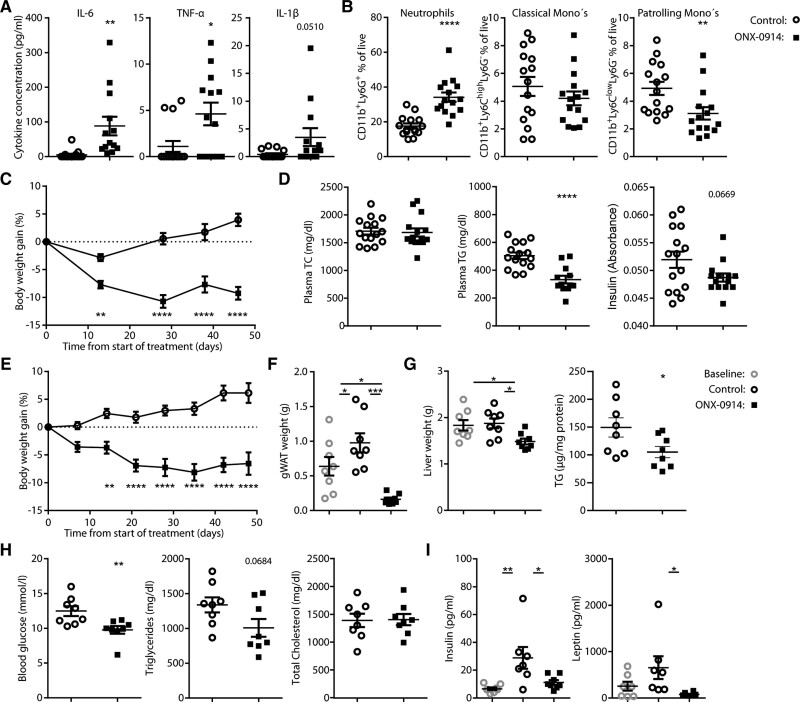
**ONX-0914 induces beneficial metabolic effects and signs of innate inflammation. A** through **D**, Female LDLr^–/–^ (n=15) were treated with ONX-0914 (10 mg/kg, 3× weekly intraperitoneally) or control-treated for 7 weeks, although fed a Western-type diet (WTD). **A**, Quantification of cytokine levels in blood plasma and (**B**) most abundant myeloid cell populations in circulation determined by flow cytometry at sacrifice. **C**, Body weight gain over the course of the experiment. **D**, Total cholesterol (TC), triglyceride (TG), and insulin levels in blood plasma at sacrifice. **E** through **I**, Male LDLr^–/–^ mice (n=8) were fed a WTD for 4 weeks after which a baseline group was euthanized. The remaining mice were treated for 7 weeks with ONX-0914 (10 mg/kg, 3× weekly intraperitoneally) or control-treated (4% DMSO in PBS). **E**, Body weight gain. **F**, Perigonadal white adipose tissue (gWAT) and (**G**) liver weight and liver TG content after sacrifice. **H**, Blood glucose, TG, and total cholesterol levels in blood plasma after 4-hour fasting after 3.5 weeks of treatment. **I**, Insulin and leptin concentrations in serum at sacrifice determined with multiplex ELISA. Expressed as mean±SEM, *P<0.05, **P<0.01, ***P<0.001, ****P<0.0001 (**A**, **B**, **D**, **G**: graph 2; **H**: Student *t* test; **C** and **E**: 2-way repeated-measures ANOVA with Šídák post hoc test; **G** and **I**: 1-way ANOVA with Šídák post hoc test). IL indicates interleukin; and TNF, tumor necrosis factor.

Since ONX-0914 is known to suppress proinflammatory cytokine levels,^11,12^ we assessed the serum cytokine profile in the serum of ONX-0914–treated mice using multiplex ELISA. Unexpectedly, elevated levels of IL-6 and TNF-α (tumor necrosis factor-α), and a trend toward increased IL-1β levels were detected in the serum of ONX-0914–treated mice (Figure 5A). We observed a significant increase in neutrophil levels in blood (Figure 5B), indicative of an ongoing innate response. Circulating classical monocyte levels remained stable, whereas patrolling monocyte populations were reduced (Figure 5B). Interestingly, ONX-0914 treatment reduced body weight (Figure 5C), virtually depleting gWAT (visual observation), and lowered plasma triglyceride levels but not plasma cholesterol levels (Figure 5D). As C57BL/6J mice on a WTD are prone to develop adiposity-driven insulin resistance,^29,30^ we assessed insulin levels in the blood, which showed a trend toward a reduction upon immunoproteasomal inhibition (Figure 5D).

Because lowered body weight and improved markers of metabolic syndrome as a result of ONX-0914 treatment have not been described previously, we further investigated the effects of immunoproteasomal inhibition on energy metabolism. To assess the effect of ONX-0914 on preexisting fat mass, male LDLr^–/–^ mice were fed a WTD for 4 weeks, upon which mice were randomized based on body weight and age. A baseline group was euthanized, while the remaining animals were vehicle-treated or ONX-0914 treated for 7 weeks. ONX-0914 led to a decline in body weight from the start of treatment up to 4 to 5 weeks of treatment after which body weight stabilized (Figure 5E).

gWAT weight increased in the vehicle-treated group compared with baseline and was reduced in ONX-0914–treated animals compared with baseline (Figure 5F). Liver weight and liver triglyceride levels were reduced upon ONX-0914 treatment, indicative of reduced steatosis in ONX-0914–treated animals (Figure 5G, representative micrographs in Figure S7A). After 4 weeks of treatment and 40 hours after the last ONX-0914 administration, fasted blood glucose, plasma triglyceride, and plasma cholesterol levels were determined. Blood glucose was significantly lower, and a trend toward reduced triglyceride levels was found in the ONX-0914–treated group compared with the vehicle-treated group while total cholesterol levels were not affected (Figure 5H). Furthermore, multiplex analysis of metabolism-related hormone levels in serum collected at sacrifice revealed increased insulin levels in the vehicle-treated group, suggestive of insulin resistance, while insulin levels were similar to baseline in ONX-0914 treated animals (Figure 5I). Leptin levels, correlating with gWAT weight, were lowered in ONX-0914 treated animals compared with vehicle-control group (Figure 5I).

To assess glucose and insulin sensitivity, GTT and ITT were performed in male LDLr^–/–^ mice fed a WTD for 10 weeks and treated with ONX-0914 or control for the final 3 weeks. Fasting blood glucose levels were again lower in the ONX-0914–treated group compared with the control group (Figure S8A). We did not observe a difference in the clearance of the oral glucose bolus (Figure S8B), or glucose levels after insulin administration before glucoregulatory compensatory mechanisms were activated (Figure S8C). However, during the GTT, glucose levels remained lower in the ONX-0914–treated group compared with the control group (Figure S8B), indicating a lowered glucose set point due to ONX-0914 treatment.

To confirm the weight loss was not due to an off-target (hepato)toxic effect of ONX-0914 treatment, we determined the activity of the liver-derived enzymes ALT (alanine aminotransaminase) and AST (aspartate aminotransferase) in blood serum, and hepatic gene expression of Cyp3A11, as a readout for PXR (pregnane x receptor) activation. We did not observe differences in ALAT and ASAT activities (Figure S9A) nor in the expression of Cyp3A11 (Figure S9B) between the vehicle and treated group, indicating that ONX-0914 treatment did not induce liver toxicity or PXR activation.

### ONX-0914–Mediated Reduction of White Adipose Tissue Mass Is Accompanied by Macrophage Accumulation in Visceral White Adipose Tissue Depots

In humans, a homozygous missense mutation in PSMB8 (proteasome subunit beta type-8), encoding for LMP7, causes lipodystrophy, adipose inflammation, and enhanced IL-6 levels.^31^ Since this phenotype is remarkably similar to the phenotype we observe upon LMP7/2 inhibition in mice, we assessed whether a reduced white adipose tissue (WAT) mass was accompanied by macrophage infiltration in perigonadal (Figure S10A), retroperitoneal (Figure S10B), perirenal (Figure S10C), and mesenteric (Figure S10D) visceral WAT depots after treatment with ONX-0914 for 3 weeks. In the perigonadal and retroperitoneal WAT depots, Cd68, Arg1 (arginase-1), Il10, Tnf (trend in gWAT), and Il1b were significantly increased in the ONX-0914–treated group compared with control-treated, while Nos2 and Il6 did not differ significantly between the treatment groups. Effects of ONX-0914 treatment were less pronounced in the perirenal and mesenteric WAT depots, showing only trends in increased gene expression of Il10, Il6, Tnf, and Il1b. These data confirm an increased M2-like macrophage infiltrate in the perigonadal and retroperitoneal WAT depots and implicate that the WAT depots could be the source of the increased proinflammatory plasma cytokines in ONX-0914–treated mice (Figure 5A).

**Figure 6.**
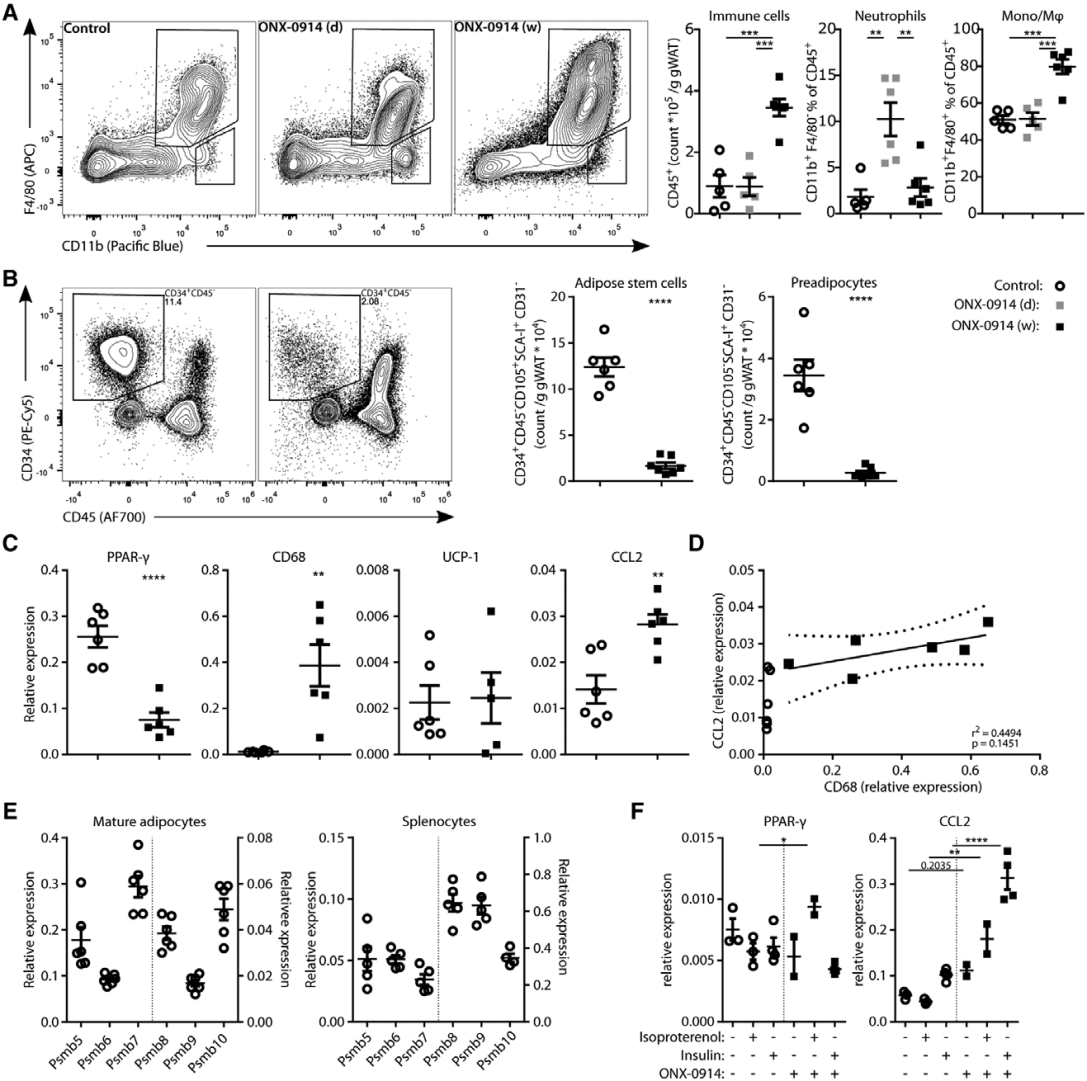
**LMP7 (proteasome subunit β5i/large multifunctional peptidase 7) inhibition induces CCL2 (CC chemokine ligand 2) expression in perigonadal white adipose tissue (gWAT) and mature adipocytes and promotes innate immune infiltrate. A**, Female LDLr^–/–^ mice (n=5–6) were fed a Western-type diet for 6 weeks, after which they were treated for 1 week (4 ONX-0914 injections) or 1 day (3 control injections followed by 1 ONX-0914 injection), or with vehicle injections (4 control injections). Representative flow cytometry plots of the CD45^+^ immune fraction of the gWAT stromal vascular fraction (SVF), showing the gating and quantifications for the CD11b^+^F4/80^+^ macrophages and CD11b^+^F4/80^−^ neutrophils. **B** through **E**, Female LDLr^–/–^ were fed a Western-type diet (WTD) for 27 weeks and treated with ONX-0914 or control in the last week of WTD feeding before sacrifice. **B**, Representative flow cytometry plots of the live singlet population from the gWAT SVF, depicting the gating of CD34^+^CD45^−^ cell population, which contains the quantified adipose stem cell and preadipocyte populations. **C**, Gene expression of the floating cell fraction from the digested gWAT. **D**, Correlation analysis between CD68 and CCL2 expression of the floating cell fraction, showing the correlation statistics of the ONX-0914–treated group. **E**, Gene expression of control-treated mice of proteasomal active subunits in freshly isolated mature adipocytes and splenocytes from the control-treated mice. **F**, Gene expression of mature adipocytes of untreated LDLr^–/–^ mice, cultured ex vivo without or with ONX-0914 (200 nmol/L) for 24 hours under basal, lipolytic (10-µmol/L isoproterenol), and lipogenic (100 nmol/L insulin) conditions. Expressed as mean±SEM, *P<0.05, **P<0.01, ***P<0.001, ****P<0.0001. **A**: One-way ANOVA with Šídák post hoc test; **B** and **C**: Student *t* tests; **F**: 2-way ANOVA (condition: control, isoproterenol, and insulin; treatment: control and ONX-0914) with Šídák post hoc test.

To further assess the kinetics of macrophage accumulation in gWAT, mice were fed a WTD for 6 weeks to induce adiposity and then treated with ONX-0914 for 1 week, 1 day, or with vehicle injections. Eighteen hours after the final injection, mice were euthanized and the SVF of the gWAT, comprised of cells derived from the vasculature, immune cells, and precursors of mature adipocytes, was isolated and assessed by flow cytometry. A single injection of ONX-0914 led to increased levels of CD11b^+^F4/80^−^ cells in the SVF, most likely representing neutrophils (Figure 6A). A week of ONX-0914 treatment led to increased overall immune cell counts (CD45^+^CD34^−^) and accumulation of CD11b^+^F4/80^+^ monocytes/macrophages in the SVF (Figure 6A), confirming the infiltration of immune cells in the perigonadal white adipose tissue upon ONX-0914 treatment.

Since LMP7 deficiency was previously found to reduce preadipocyte differentiation into mature adipocytes,^31,32^ we then quantified the number of adipose stem cells and preadipocytes in the SVF of mice on WTD for 27 weeks, treated with ONX-0914 in the last for a week by flow cytometry. LMP7/2 inhibition massively reduced the number of adipose stem cells and committed preadipocytes in the stromal fraction (Figure 6B).

To assess whether ONX-0914 directly affected the mature adipocyte population, RNA was isolated from the adipocyte cell fraction obtained from gWAT after 1 week of treatment with ONX-0914. UCP-1 expression was not increased (Figure 6C), suggesting that ONX-0914 treatment did not induce the beiging of white adipose tissue, a process in which white adipocytes obtain thermogenic capabilities associated with improved glucose and lipid metabolism and enhanced energy expenditure.^33,34^ Surprisingly, the expression of PPAR-γ (peroxisome proliferator-activated receptor), a marker of mature adipocytes, was significantly decreased by 3-fold with a concomitant increase in CD68 and CCL2 (CC chemokine ligand 2) expression (Figure 6C), indicating the presence of macrophages in the adipocyte cell fraction. Correlation analysis of CCL2 and CD68 expression of the ONX-0914–treated group suggested that CCL2 expression was unlikely to be solely derived from macrophages (r^2^=0.45; *P*=0.15; Figure 6D). Freshly isolated primary mature adipocytes expressed immunoproteasomal catalytic subunits (Figure 6E), suggesting that ONX-0914 could also directly affect adipocytes, although expression levels were relatively low compared with the expression of constitutive subunits and expression of immunoproteasomal subunits in splenocytes (Figure 6E). Primary mature adipocytes cultured in lipogenic (insulin) or lipolytic (isoproterenol) conditions indeed upregulated CCL2 expression upon overnight coincubation with ONX-0914 (200 nmol/L; Figure 6F). The ONX-0914–mediated CCL2 production in mature adipocytes could therefore underlie the innate infiltrate of the gWAT upon ONX-0914 treatment.

### ONX-0914 Reduces Intestinal Lipid Uptake and Gastric Emptying

To further investigate the mechanism of ONX-0914–mediated reduction of gWAT mass, we assessed whether LMP7/2 inhibition affected energy expenditure using metabolic cages. Since triglyceride levels were lowered by LMP7/2 inhibition, we also studied the effect of ONX-0914 on clearance of IV-injected VLDL-like particles.^35^ Because our initial studies in LDLr^–/–^ mice may have obscured a possible cholesterol-lowering effect of ONX-0914 due to the absence of a functional LDLr,^33^ we used E3L.CETP hyperlipidemic mice in this experiment. After 3 weeks of WTD feeding, ONX-0914 treatment was given for 2.5 weeks. Also, in the E3L.CETP mice, ONX-0914 induced macrophage accumulation in perigonadal (Figure S11A), but not in mesenteric (Figure S11B) and subcutaneous (Figure S11C) WAT depots, while inducing an M2-like macrophage phenotype (Figure S11A through S11C).

**Figure 7.**
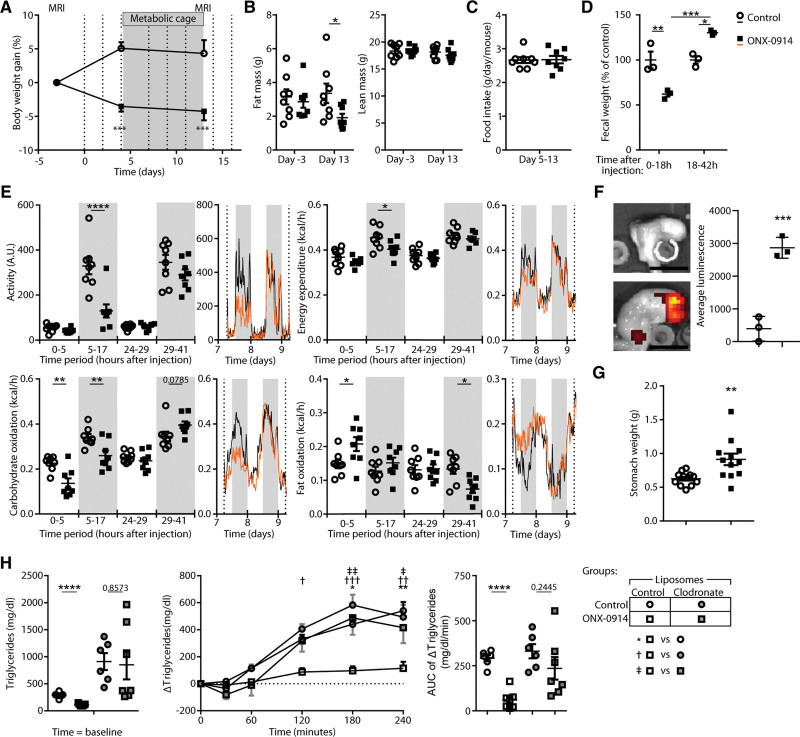
**Reduced gastric emptying and intestinal lipid uptake in ONX-0914–treated mice. A** through **E**, Female E3L.CETP mice (n=8) were fed a Western-type diet (WTD) for 3 weeks after which they were treated with ONX-0914 (10 mg/kg, 3× weekly, intraperitoneally) or control-treated (4% DMSO in PBS) for 2.5 weeks. **A**, The experimental set up depicting the EchoMRI time points at 3 days before treatment and day 13 of treatment, the injection times depicted by dotted lines, the time span mice were monitored in metabolic cages, and body weight at randomization and measured during the study. **B**, The fat mass and lean mass as determined by EchoMRI. **C**, The average food intake during days 5 to 13. **D**, Feces was collected from the bedding from cages housing multiple animals at days 14 to 15 and weighed and normalized for the number of mice per cage. **E**, Activity, energy expenditure, glucose utilization, and lipid utilization as determined in metabolic cages. **F**, Male LDLr^–/–^ mice (n=3) were fed a WTD for 3 weeks, after which they were treated with ONX-0914 for a week. Mice were starved for 5 hours after which GastroSense 750 was administered by oral gavage. Thirty minutes after oral gavage, luminescence in the intestinal tract was assessed with IVIS. **G**, Stomach weight of LDLr^–/–^ (n=12) mice fed a WTD for 6 weeks, after which they were treated with ONX-0914 for 6 weeks while on a standard laboratory diet. **H**, Female LDLr^–/–^ mice (n=8) were fed a WTD for 27 weeks after which they were treated with ONX-0914 (10 mg/kg, 3× weekly, intraperitoneally) or control-treated (4% DMSO in PBS) for 1 week. A day before ONX-0914 treatment, half of the mice received clodronate liposomes or control liposomes. After final ONX-0914 injection mice were starved overnight. Thereafter, baseline triglyceride (TG) levels were determined in blood plasma and mice received an oral bolus of olive oil (200 µL), after which TG levels were collected at the indicated time points to determine the change in TG concentration, and the area under the curve of the change in TG levels in the blood plasma. Expressed as mean±SEM, *P<0.05, **P<0.01, ***P<0.001, ****P<0.0001, **A**, **B**, **D**, **E**, **H** (**middle**): 2-way repeated-measures ANOVA with Šídák post hoc test; **F** and **G**: Student *t* test, **H** (**left** and **right**): 2-way ANOVA with Šídák post hoc test. k post hoc test; **F** and **G**: Student *t* test, **H** (**left** and **right**): 2-way ANOVA with Šídák post hoc test.

Two weeks of treatment of E3L.CETP mice with ONX-0914 reduced body weight gain compared with the control treatment (Figure 7A). Similar to the studies using LDLr knock-out mice, ONX-0914 reduced fat mass but not lean mass as assessed by EchoMRI (Figure 7B), while overall food intake was similar (Figure 7C). Unexpectedly, locomotor activity and energy expenditure were decreased during the dark phase after ONX-0914 treatment (Figure 7E). Carbohydrate oxidation decreased directly after ONX-0914 treatment and was accompanied by increased fat oxidation after treatment (Figure 7E). The night after treatment (29–41 hours after treatment) locomotor activity normalized and fat oxidation returned to control level while carbohydrate oxidation tended to increase (Figure 7E), suggestive of reduced food intake directly after treatment and subsequent compensational feeding in the next 24 hours. In line with a shifted feeding pattern due to ONX-0914 treatment, the fecal output was reduced in the first 18 hours and increased in the 18- to 42-hour time frame after ONX-0914 treatment (Figure 7D). Triglyceride and total non-HDL cholesterol levels were lowered by LMP7/2 inhibition (Figure S12A and S12B); however, the clearance rate of [^14^C]cholesteryl oleate and glycerol tri[^3^H]oleate from IV-injected VLDL-like particles was slightly slower in ONX-0914–treated animals (Figure S12C), indicating that lowered triglyceride and total cholesterol levels were not due to a faster uptake from the blood.

As intestinal macrophages play an important role in gastrointestinal homeostasis, we assessed whether ONX-0914 treatment reduced gastrointestinal emptying and intestinal triglyceride uptake, which could explain the altered feeding pattern and the reduced body weight after ONX-0914 treatment. An increase in luminescence was measured in the stomachs of ONX-0914–treated mice 30 minutes after oral gavage of a fluorescent agent (Figure 7F), and an increase in stomach weight was observed in ONX-0914–treated mice (Figure 7G), both supporting that ONX-0914 treatment can inhibit gastric emptying.

To assess intestinal lipid uptake, an oral lipid loading test was performed. To determine the involvement of macrophages in this process, the majority of intraperitoneal macrophages were depleted with intraperitoneal injections of clodronate liposomes a day before control or ONX-0914 treatment and compared with mice injected with empty liposomes. Macrophage and neutrophil numbers were increased in gWAT after a week of ONX-0914 treatment as previously (Figure S13A through S13D). Clodronate liposome treatment successfully reduced CD11b^+^F4/80^+^CD206^+^ adipose tissue–resident macrophage levels (Figure S13A and S13B) and reduced accumulation of CD206^−^ macrophages (Figure S13A and S13B) and neutrophils (Figure S13C and S13D) after ONX-0914 treatment. After 3 ONX-0914 injections, mice were fasted overnight after which an oral oil load test was performed. Macrophage depletion increased baseline triglyceride levels in line with previous data,^36^ while only in the control liposome/ONX-0914–treated group, triglyceride uptake was decreased, suggesting that ONX-0914 acted through a macrophage population to reduce intestinal lipid uptake (Figure 7H). In LMP7-deficient animals, reduced intestinal lipid uptake was reported to be the result of a lower expression of fat-digesting lipases, Pnlip (pancreatic lipase), and Pnliprp2 (pancreatic lipase-related protein 2)-like^37^; however, we did not observe this (Figure S14A) nor did we observe reduced expression of proteins involved in intestinal lipid uptake in the intestines after ONX-0914 treatment (Figure S14B). Furthermore, reduced intestinal lipid uptake was not caused by intestinal inflammation as macrophage (Figure S15A), T cell (Figure S15B), and inflammatory cytokine gene expression in the intestines (Figure S15C) were unaffected upon ONX-0914 treatment.

To find an explanation for the observed metabolic phenotype, we consulted literature for an ER stress–inducible cytokine with weight-lowering effects. As GDF15 (growth differentiation factor 15) fits this description and is produced by adipose tissue macrophages (among other cell types) and has also immunoinhibitory effects,^38–44^ we assessed the GDF15 levels in the atherosclerosis initiation study (female LDLr^−/−^ fed a WTD and treated for 7 weeks), preexisting adipose tissue study (male LDLr^−/−^ mice on a WTD for 11 weeks and treated the last 7 weeks), and short-term treatment study (male LDLr^−/−^ on a WTD for 5 weeks, treated for 1 day or 1 week). ONX-0914 treatment upregulated blood plasma GDF15 concentrations in all of these studies (Figure S16A through S16C). The induction of GDF15 was rapid, as already after 16 hours of a single dose of ONX-0914 enhanced levels of GDF15 were observed (Figure S16C). Furthermore, GDF15 levels in the ONX-0914–treated groups appeared variable depending on the time point of blood collection (Figure S16A through S16C), fitting with a reduced metabolic effect upon prolonged ONX-0914 treatment. The ONX-0914–mediated induction of GDF15 might have contributed to the metabolic effects of ONX-0914.

## DISCUSSION

The inflammatory response in atherosclerosis is marked by a profound Th1 response,^2,45^ resulting in the production of the atherogenic Th1 cytokines IFN-γ^46–50^ and TNFα.^51,52^ Because of the detrimental effects of the Th1 response in atherosclerosis,^2,53,54^ we aimed to inhibit this response by using ONX-0914, an immunoproteasomal inhibitor, which was previously described to inhibit Th1 and Th17 development,^9,11^ reduce secretion of proinflammatory cytokines,^9,55^ and reduce various autoimmune diseases in experimental models.^9–12,14–18^ In line with results from Liao et al,^19^ we show that ONX-0914 treatment reduces atherosclerosis in LDLr^–/–^ mice on a WTD. Although Liao et al^19^ dedicate the effect of ONX-0914 to increased efferocytosis upon immunoproteasomal inhibition, we find numerous additional beneficial immune effects of ONX-0914 treatment in the context of initial and advanced atherosclerosis in LDLr^–/–^ mice, as well as substantial improvements in markers of metabolic syndrome in LDLr^–/–^ and E3L.CETP mice.

We show that immunoproteasomal inhibition can effectively inhibit the pathogenic T cells in the context of atherosclerosis. In our early atherosclerosis model, we reveal that immunoproteasomal inhibition can reduce CD4^+^ and CD8^+^ Tem and Tcm cell populations in several immune compartments. In our models of advanced atherosclerosis, ONX-0914 treatment also rapidly reduced the levels of Th1 cells and reduced T cell proliferation. This is of particular interest as in humans, circulating Tem cells correlate with carotid intima-media thickness independent of classical cardiovascular disease risk factors and are increased in patients with stable angina and acute myocardial infarction compared with healthy controls.^56^

The reduction in memory T cells could be a direct effect of immunoproteasomal inhibition^11^ but might also be a consequence of the observed reduction in levels and activation of APCs upon ONX-0914 treatment. This would be in line with the finding that immunoproteasome-deficient DCs are inferior at priming T cell responses compared with wild-type DCs.^13^ Although (tolerogenic) DCs can also induce favorable immune responses toward (ox)LDL,^57,58^ MHC-II–dependent activation of proinflammatory CD4^+^ T cell specific for ApoB100 promotes atherosclerosis.^59^ Similarly, enhanced activation of DCs increased CD4^+^ and CD8^+^ T cell activation and is proatherogenic.^26^ Therefore, the reduced levels and activation of DCs that we find upon ONX-0914 treatment likely contributed to the smaller lesions observed after ONX-0914 treatment. The M2-like macrophage phenotype we observe in adipose tissue, and reduced proinflammatory markers on macrophages in the plaque and spleen upon ONX-0914 treatment are in line with the augmented alternative polarization of macrophages due to immunoproteasome dysfunction,^60^ also known to inhibit atherosclerosis.^27,28^

The precise molecular mechanisms underlying the impact of immunoproteasomal inhibition on immune cell inhibition are not entirely elucidated. Nonetheless, it is likely that at least in part these immunosuppressive effects are mediated through the unfolded protein response (UPR) pathway. The UPR is initiated in response to endoplasmic reticulum (ER) stress, which can be triggered by the accumulation of improperly folded and unfolded proteins, previously reported in various immunoproteasome inhibitor–treated immune cells.^61,62^ The UPR involves the activation of specific genes responsible for mitigating ER stress and restoring protein homeostasis and can induce apoptosis when the ER stress exceeds a cell’s capacity to cope.^63^ Through stabilization of Nrf2 (nuclear factor erythroid–derived 2-related factor 2), the UPR induces the upregulation of constitutive proteasomes, which we observe in the spleen and cultured DCs upon ONX-0914 treatment. Additionally, Nrf2 has been demonstrated to suppress the activity of immune cells.^64–67^ Concomitant with the induction of the constitutive proteasome, the immunoproteasomes were downregulated in the spleen and BMDCs, possibly resulting from a general reduction in protein synthesis because of UPR activation. However, the reduced inflammatory status of immune cells treated with ONX-0914 could have contributed to the lowered immunoproteasome expression, as immunoproteasomes are generally upregulated upon immune cell activation, aided by the presence of NF-κb and IFN-responsive elements in the promoters of the immunoproteasomal catalytic subunits.^68–70^ Through this inhibition of immune cells with disturbed proteostasis, the UPR prevents tissue damage by unfit immune cells.

We show that ONX-0914 consistently reduced body weight in both WTD-fed LDLr^–/–^ mice and E3L.CETP mice. WAT mass was markedly decreased while lean mass was preserved, concurrent with improved metabolic parameters such as lower insulin levels, fasting blood glucose, triglyceride levels, and E3L.CETP mice also lower cholesterol levels. To our knowledge, this is the first study to show that LMP7/2 inhibition using the specific inhibitor ONX-0914 has metabolic effects in addition to its immunomodulatory effects. These effects were potentially overlooked in previous studies in which the experimental design was based on lean mice on a standard laboratory diet rather than models of dyslipidemia and atherosclerosis.^9–12,14–18^ Off target activation of PXR or (hepato)toxicity did not underlie the metabolic effects of ONX-0914 treatment.

Interestingly, patients with a loss of function mutation in PSMB8, coding for LMP7, present with similar symptoms as described in animal studies including elevated serum IL-6 levels, white adipose tissue inflammation, and lipodystrophy.^31^ In our investigation, we have identified that mature adipocytes derived from perigonadal white adipose tissue express immunoproteasomal catalytic subunits and increase the production of CCL2 in response to exposure to ONX-0914, thereby establishing a direct mechanism through which ONX-0914 induces innate immune cell infiltration in perigonadal white adipose tissue, and likely also retroperitoneal adipose tissue. The macrophages in the WAT depots appeared to be have a more M2-like phenotype upon immunoproteasomal inhibition, as was previously described in alveolar macrophages.^60^

Adipose tissue inflammation is classically associated with insulin resistance; however, we do not observe our experimental models following ONX-0914 treatment. Recent experimental evidence suggests that insulin resistance may precede adipose tissue inflammation, implying that the initiation of insulin resistance does not hinge on adipose tissue inflammation.^71^ In fact, signaling mediated by TNF-α and IL-1β is indispensable for the expansion of adipose tissue, and the absence of this proinflammatory signaling exacerbates insulin resistance in diet-induced obese mice.^72^ These findings underscore the crucial role that certain inflammatory mediators play in maintaining healthy white adipose tissue homeostasis. In line with these recent findings, therapies aimed at blocking systemic TNF-α and IL-1β do not ameliorate insulin resistance in patients.^73,74^ Thus, the relationship between inflammation in white adipose tissue and insulin resistance appears to be more intricate than the conventional paradigm of adipose tissue inflammation causing insulin resistance. It is plausible that the impact of inflammation is interdependent with other environmental factors, such as tissue hypoxia, which occurs when adipose tissue undergoes expansion.

LMP7/2 inhibition reduced the increase in blood plasma triglyceride during an oral lipid loading test, suggesting that the intestinal uptake of lipids was reduced by LMP7/2 inhibition, which was also reported in LMP7^–/–^ mice.^37^ We found that ONX-0914–mediated inhibition of increased triglyceride levels in blood plasma during the oral lipid loading test was macrophage dependent since the phenotype was lost when peritoneal macrophages were depleted with clodronate liposomes. In LMP7^–/–^ mice, reduced intestinal lipid absorption was speculated to be due to a reduction in pancreatic lipases Pnlip and Pnlrp2^37^; however, pharmacological inhibition of LMP7/2 did not affect the expression of Pnlip and Pnlrp2 in our study. Because intestinal lipid uptake is also minimally affected in Pnlip-deficient mice^75^ and Pnlrp2^76^-deficient mice, the cause of reduced intestinal lipid uptake upon decreased immunoproteasome activity is possibly (co-)dependent on other mechanisms. In our study, LMP7/2 inhibition also reduced gastric emptying. Therefore, we cannot exclude that the reduced increase in blood plasma triglyceride in the oral lipid loading test is entirely, or partly due to this delay in gastric emptying. Delayed gastric emptying is known to reduce caloric intake due to enhanced satiety,^77^ which might underlie the reduced food intake the day after ONX-0914 injection and the compensatory feeding observed the second day after ONX-0914 injection in the metabolic cage study.

Our data suggest that peritoneal macrophages have a central role in the ONX-0914–mediated metabolic effects. Various factors produced by macrophages, including TNF-α,^78^ IL-6,^79^ IL-1β,^80^ IL-15, and GDF15,^81^ are known to affect metabolism. Especially, GDF15, which is also induced by the UPR^38^ and expressed in high levels by adipose tissue macrophages,^39^ has been the focus of recent investigations for its capacity to regulate body weight. Via signaling through GFRAL (GDNF [glial-derived neurotrophic factor] receptor alpha-like) receptor-expressing neurons in the hindbrain GDF15 reduces gastric emptying,^40^ induces taste aversion,^41^ and reduces food intake,^40–42^ which could explain the altered feeding pattern we observe upon ONX-0914 treatment. Furthermore, GDF15 was reported to enhance lipolysis and thermogenesis without a significant reduction in food intake through activation of the sympathetic nervous system,^39^ which could also have contributed to ONX-0914–mediated reduction in body weight.^43^ Through a least partially adiposity reduction unrelated and yet unknown mechanisms, GDF15 also reduced circulating glucose and insulin levels, and improved glucose tolerance and insulin responsiveness,^44^ in line with the lowered glucose and insulin levels we observed in ONX-0914–treated mice, although we could not confirm enhanced glucose uptake or insulin sensitivity in GTT and ITT assays.

In conclusion, treatment of mice with ONX-0914 reduces atherosclerosis and considerably reduces WAT mass in obese mice fed a WTD, concomitantly improving the parameters of metabolic syndrome. Because atherosclerosis is still the primary cause of death worldwide, and the obesity epidemic is feeding metabolic syndrome–related diseases, immunoproteasomal inhibition may be a valuable pharmacological tool to combat both. First data from clinical trials with immunoproteasomal inhibitor Zetomipzomib (KZR-616)^82^ indicate that immunoproteasomal inhibition is safe, well tolerated, and effective in patients with systemic lupus erythematosus.^83^ It would be exciting to investigate whether immunoproteasomal inhibition also decreases metabolic and cardiovascular disease risk in humans.

## ARTICLE INFORMATION

### Sources of Funding

This work was supported by the European Union’s Seventh Framework (603131 to J. Kuiper, H. Douna, and F.H. Schaftenaar) funded by contributions from Academic partners; the Netherlands Cardio Vascular Research Initiative: the Dutch Heart Foundation, Dutch Federation of University Medical Centers, the Netherlands: Organisation for Health Research and Development and the Royal Netherlands Academy of Sciences’ for the GENIUS-II project Finding new targets for diagnosis and treatment of atherosclerosis (CVON2017-2020 to J. Kuiper, P.C.N. Rensen, and F.H. Schaftenaar); the Netherlands Heart Foundation (2016T008 and 2018T051 to A.C. Foks, 2019T067 to I. Bot, and 2009T038 to P.C.N. Rensen).

### Disclosures

None.

### Supplemental Material

Table S1

Figures S1–S16

Gating Strategies

Major Resources Table

## Supplementary Material
