# Supplementary material for: Immunoproteasomal Inhibition With ONX-0914 Attenuates Atherosclerosis and Reduces White Adipose Tissue Mass and Metabolic Syndrome in Mice
Source: Arterioscler Thromb Vasc Biol. 2024 Apr 25;44(6):1346–64. doi: 10.1161/ATVBAHA.123.319701 (PMC11188635; doi:10.1161/ATVBAHA.123.319701)
Supplement: Supplementary file 1 [file atv-44-1346-s001.pdf]

Supplemental Materials

Immunoproteasomal inhibition with ONX-0914 attenuates atherosclerosis and reduces white adipose tissue mass and metabolic syndrome

Authors:
Frank H. Schaftenaar[1*], Andrea D. van Dam[2], Gerjan de Bruin[3], Marie A.C. Depuydt[1], Jill de Mol[1], Jacob Amersfoort[1], Hidde Douna[1], Menno Meijer[1], Mara J. Kröner[1], Peter J. van Santbrink[1], Mireia N.A. Bernabé Kleijn[1], Gijs H.M van Puijvelde[1], Bogdan I. Florea[3], Bram Slütter[1], Amanda C. Foks[1], Ilze Bot[1], Patrick C.N. Rensen[2], Johan Kuiper[1]

Affiliations:
[1] Division of BioTherapeutics, Leiden Academic Centre for Drug Research, Leiden, The Netherlands
[2] Department of Medicine, Division of Endocrinology, and Einthoven Laboratory for Experimental Vascular Medicine, Leiden University Medical Center, Leiden, The Netherlands
[3] Leiden Institute of Chemistry, Chemical Biology, Leiden, The Netherlands

Supplemental Table 1

Table S1. List of primers used for gene expression analysis.

Gene	Protein	Fw primer	Rev primer
Arg1	Arginase-1	tggcagaggtccagaagaatgg	gtgagcatccacccaaatgacac
Cav1	Caveolin-1	actgagaagcaagtgtatgacgcgc	cagatgccgtcgaaactgtgtgtcc
Ccl2	Chemokine (C-C motif) ligand 2	ctgaagccagctctctcttcctc	ggtgaatgagtagcagcaggtga
CD3e	T-cell surface glycoprotein CD3 epsilon chain	tctgctacacaccagcctcaaa	atgaccatcagcaagcccaga
CD68	CD68	tgcctgacaagggacacttcggg	gcgggtgatgcagaaggcgatg
Clps	Colipase	ttgcacacacaaggccatggagaa	catagttggtgttggtgatggcgc
Cyp3a11	Cytochrome P450 3A11	cccacattcaccagtggaaaactcaag	cttgcctttctttgccttctgcctc
Fabp2	Fatty acid-binding protein	cacgtgtagacaatggaaaggagctga	aatcgcttggcctcaactccttcat
Il10	Interleukin-10	gggtgagaagctgaagaccctc	tggccttgtagacaccttggtc
Il6	Interleukin-6	tgagactggggatgtctgtagctcat	gttgtcaccagcatcagtcccaaga
Ly6G	Lymphocyte antigen 6G	gatggattttgcgttgctctgga	gagtagtggggcagatgggaag
Npc1l1	NPC1-like intracellular cholesterol transporter 1	ctacacggcctggtcttcct	aaggggtactgtgggcaag
Pnlip	Pancreatic triacylglycerol lipase	tgggagcatcaaggatcacagtgga	gagacagtgtgagcaggacgtcttc
Pnliprp2	Pancreatic lipase-related protein 2	atgggagcttcacaaatcacagtgca	acgtttattatgggaagaggcacggg
Pparg	peroxisome proliferator activated receptor gamma	agggcgatcttgacaggaaagacaac	aaaattcggatggccacctctttgc
Psmb10	20S proteasome subunit beta-2i	cgtctgccctttactgcccttgg	tgatcacacaggcatccacattgcc
Psmb5	20S proteasome subunit beta-5	gcctccaaactgctcgctaacatgg	gatcctgttcccctcgctgtctacg
Psmb6	20S proteasome subunit beta-1	gaatcatcattgcaggctgggaccc	tagccatagatgtacgagctcccgg
Psmb7	20S proteasome subunit beta-2	actttcttcgtccattctcagtgccc	aaccacccacagcaccattcacg
Psmb8	20S proteasome subunit beta-5i	ttccaacatgatgctgcagtaccgg	gtggaaaacatctgtcccgagagcc
Psmb9	20S proteasome subunit beta-1i	gtgccggcgtttcaccacagat	agaattttggcagctcatctcccagg
Psme1	PA28a	tttcgctttcccttcccgct	cttgctacacaggtcttcacgga
Psme2	PA28b	ggccttgctcgctttggttaag	actctctccagcaccttctcct
Slc27a4	Long-chain fatty acid transport protein 4	ctggagagctttgcacagaccttga	ctccttccgcaactctgtcttctgg
Socs3	Suppressor of cytokine signaling 3	cccaaggccggagatttcgctt	gcgggaaacttgctgtgggtga
Ucp-1	Uncoupling protein 1	ccaagctgtgcgatgtccatgtaca	aaacatgatgacgttccaggacccg
Actb	Actin beta	cttctttgcagctccttcgttgccg	aatacagcccggggagcatcgtc
RPLP0	60S acidic ribosomal protein P0	ctgagtacaccttcccacttactga	cgactcttcctttgcttcagcttt

Supplemental figures

Supplemental Figure S1

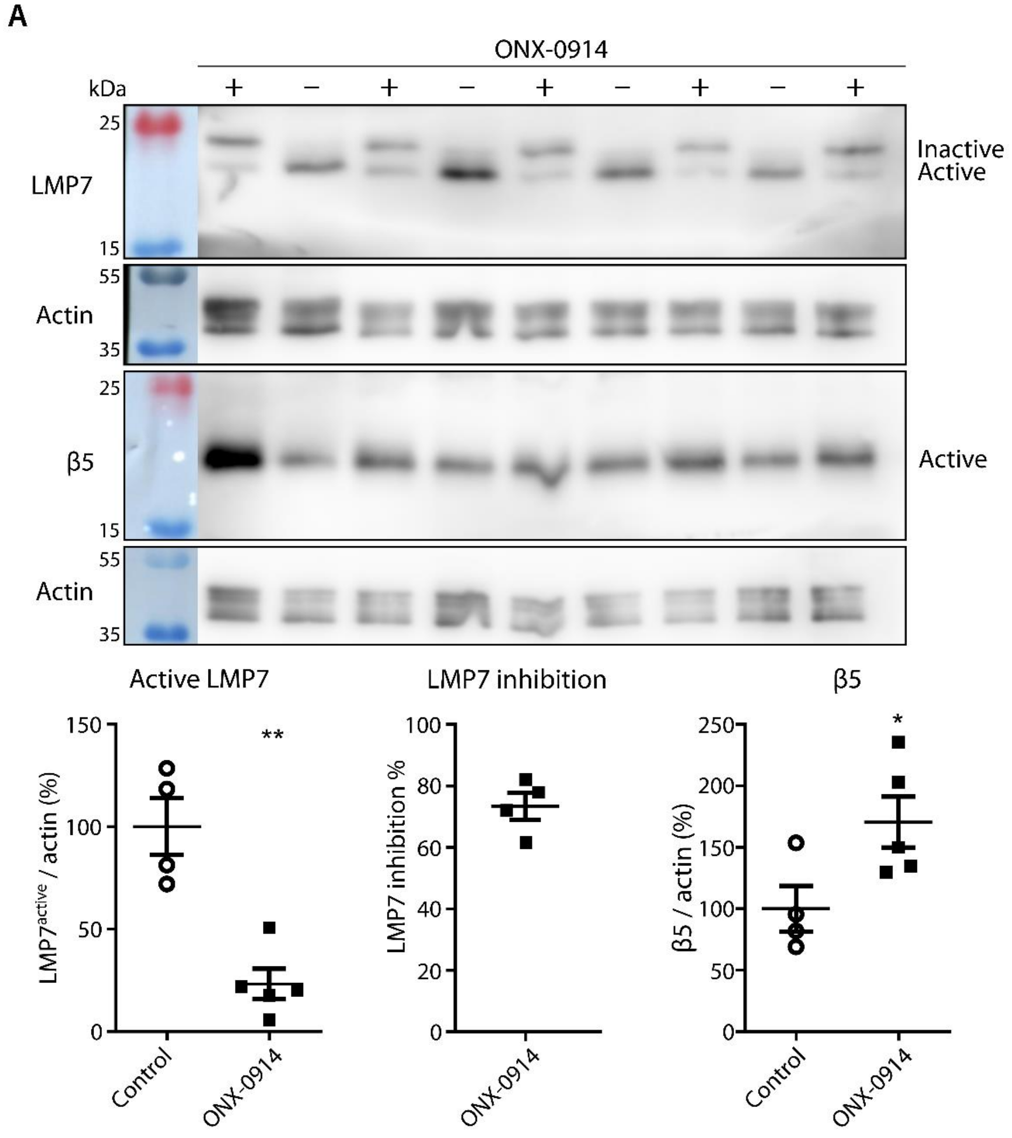

ONX-0914 treatment at 10 mg/kg specifically inhibits the immunoproteasome in vivo. (A) Western blots and quantification of β5 and LMP7 in spleen lysates from male LDLr$^{-/-}$ mice on a Western Type Diet, treated with ONX-0914 (10 mg/kg, 3 times weekly) (+) or control (-) for 1 week). Spleens were harvested 18h after final ONX-0914 administration. ONX-0914 acts through covalently binding to the active site of the catalytic subunit, resulting in an inhibited LMP7 molecule that is slightly larger than active LMP7. The intensity of the inactive LMP7 band (higher on the LMP7 western blot), and active LMP7 (lower on the LMP7 western blot) were separately quantified to estimate remaining active LMP7 (active LMP7 normalized with the actin signal, both actin bands of the top actin blot), and LMP7 inhibition (percentage of inactive LMP7 of total LMP7) upon ONX-0914 treatment. The intensity of the single β5 band (uninhibited/active) was quantified and normalized to the actin signal (both actin bands of the bottom actin blot) to estimate the relative β5 protein levels. Expressed as mean ± SEM, Unpaired T-test, $^{*}p < 0.05$, $^{**}p < 0.01$, a(graph 1 and graph 3): Student T-test.

Supplemental Figure S2

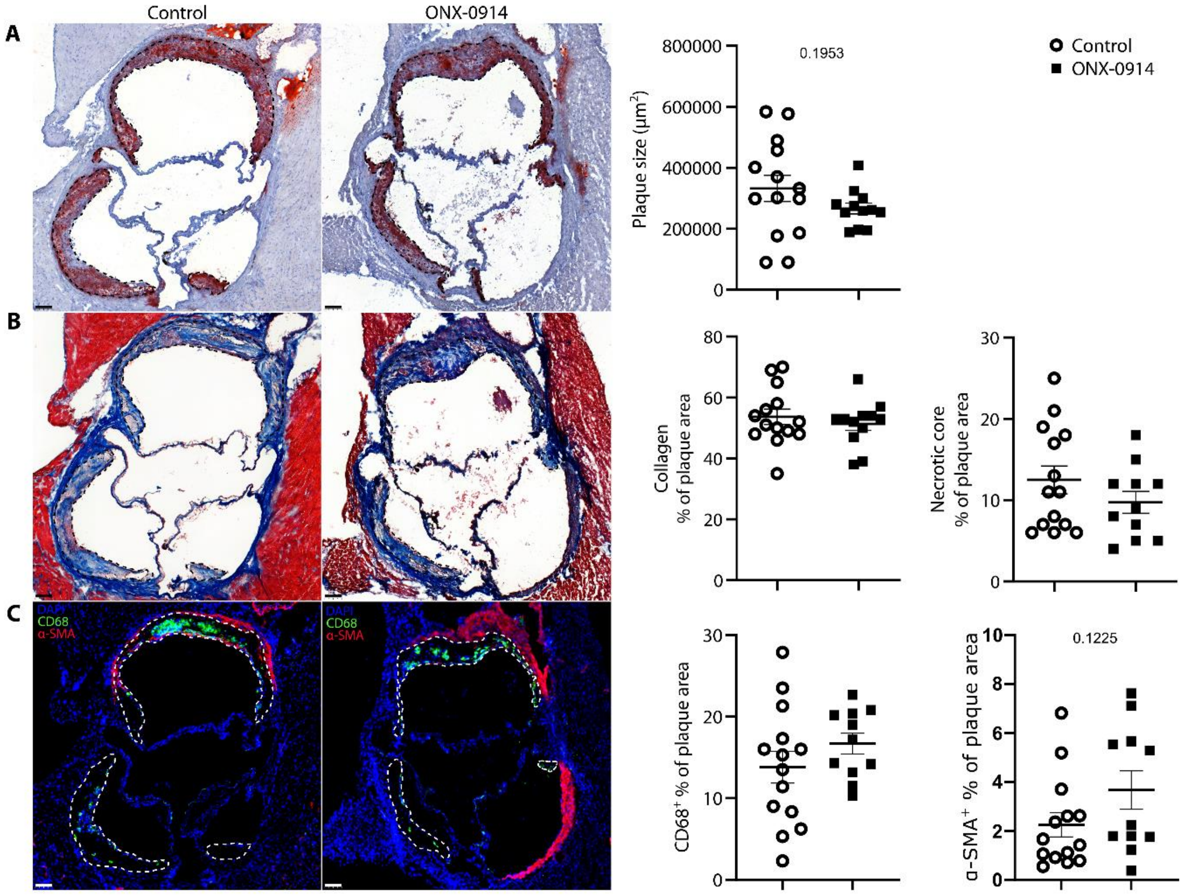

Characteristics of advanced plaques in aged mice. Representative micrographs and quantifications of histological analysis from the aortic root of aged (68-73 weeks old) male LDLr$^{-/-}$ mice (n=14-13), treated with ONX-0914 (10 mg/kg, 3 times weekly intraperitoneally) or control for 6 weeks while remaining on a chow diet, stained with (A) Oil Red O, (B) Masson's trichrome, (C) a fluorescent staining for macrophages (CD68, green), smooth muscle cells (α-SMA, red) and nuclei (DAPI, blue). Plaque tissue is outlined with a dashed line, necrotic core area is outlined with a dotted line in the Masson's trichrome micrographs. Quantifications expressed as mean ± SEM, unpaired T-test, no significant results, scale bar is 100 µm.

Supplemental figure S3

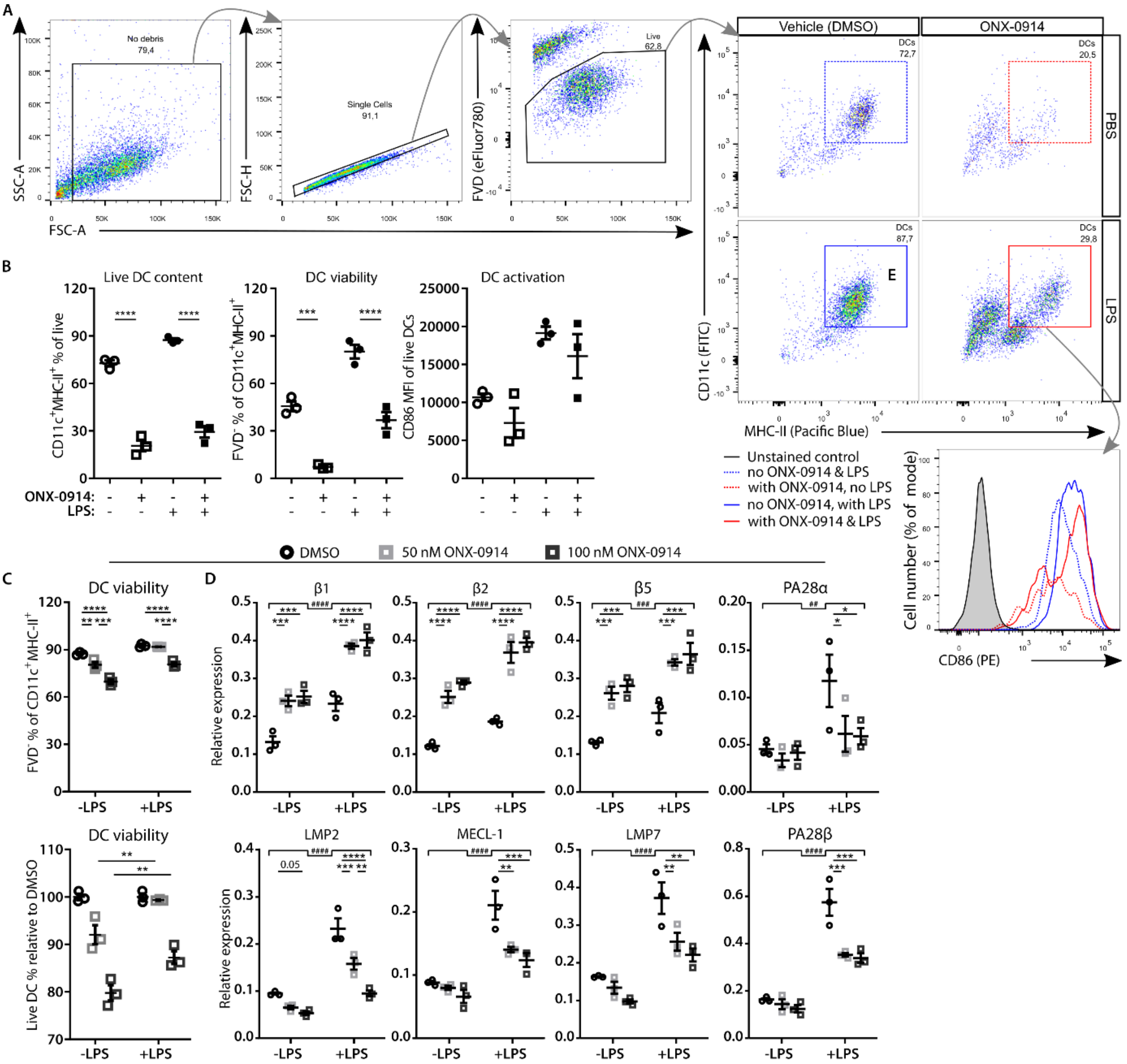

LMP7 inhibition directly inhibits DC activation and causes upregulation of constitutive proteasomal active subunits. (A) Bone marrow derived DCs derived from 8 week old male LDLr$^{-/-}$ mice were incubated with ONX-0914 (0 or 200 nM) overnight in absence or presence of LPS (100 ng/ml) and assessed by flow cytometry. (B) Cell culture viability was assessed by gating as shown in panel 'A'. For quantification of DC viability single cells were gated like in panel 'A', but thereafter CD11c^{+}MHC-II^{+} cells were gated followed by the gating of live DCs. CD86 median fluorescent intensity of the live MHC-II^{+}CD11c^{+} population was assessed as a measure for DC activation. (C) Bone marrow derived DCs were incubated with ONX-0914 (0, 50 or 100 nM) overnight in absence or presence of LPS (100 ng/ml) after which DC viability was determined by flow cytometry like explained in panel 'B', and (D) expression of catalytic constitutive and immuno- proteasomal subunits, and immunoproteasomal activators were determined by qPCR. Expressed as mean ± SEM, one-way ANOVA with Holm-Sidak posttest, *p < 0.05, **p < 0.01, *** p < 0.001 **** p < 0.0001.

Supplemental Figure S4

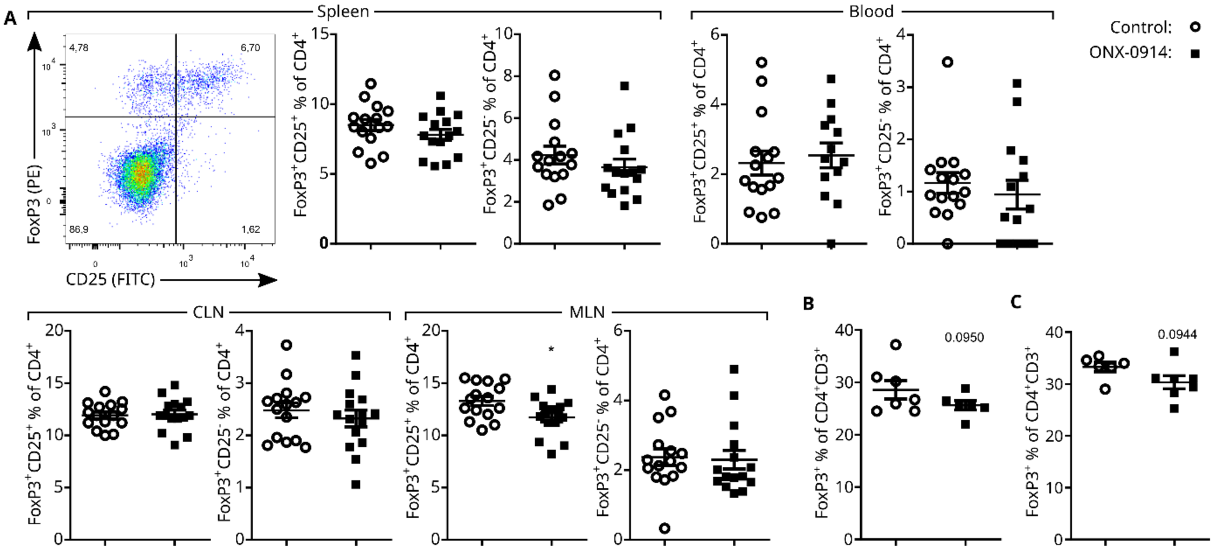

Tregs are not increased by ONX-0914 treatment in early and advanced atherosclerosis. (A) CD4$^+$ T-cell FoxP3$^+$CD25$^+$ and FoxP3$^+$CD25$^-$ populations were determined with flow cytometry in the spleen blood, cervical lymph nodes, and mesenteric lymph nodes of female LDLr$^{-/-}$ (n=15) after treatment with ONX-0914 (10 mg/kg, 3 times weekly intraperitoneally) or control treated for 7 weeks, while fed a WTD. (B) CD4$^+$FoxP3$^+$ regulatory T cell levels in the spleen of aged mice (68-73 weeks of age prior to WTD feeding) fed a WTD for 6 weeks prior to sacrifice, during which mice were treated with ONX-0914 (10 mg/kg, 3 times weekly intraperitoneally) or control, as determined by flow cytometry. (C) Female LDLr$^{-/-}$ mice (n=7) were fed a WTD for 27 weeks and treated with ONX-0914 (10 mg/kg, 3 times weekly intraperitoneally) or control in the final week of WTD, after which mice were sacrificed and CD4$^+$FoxP3$^+$ regulatory T cell levels in the spleen were determined with flow cytometry. Expressed as mean ± SEM, unpaired two tailed t-test, * $p < 0.05$.

Supplemental Figure S5

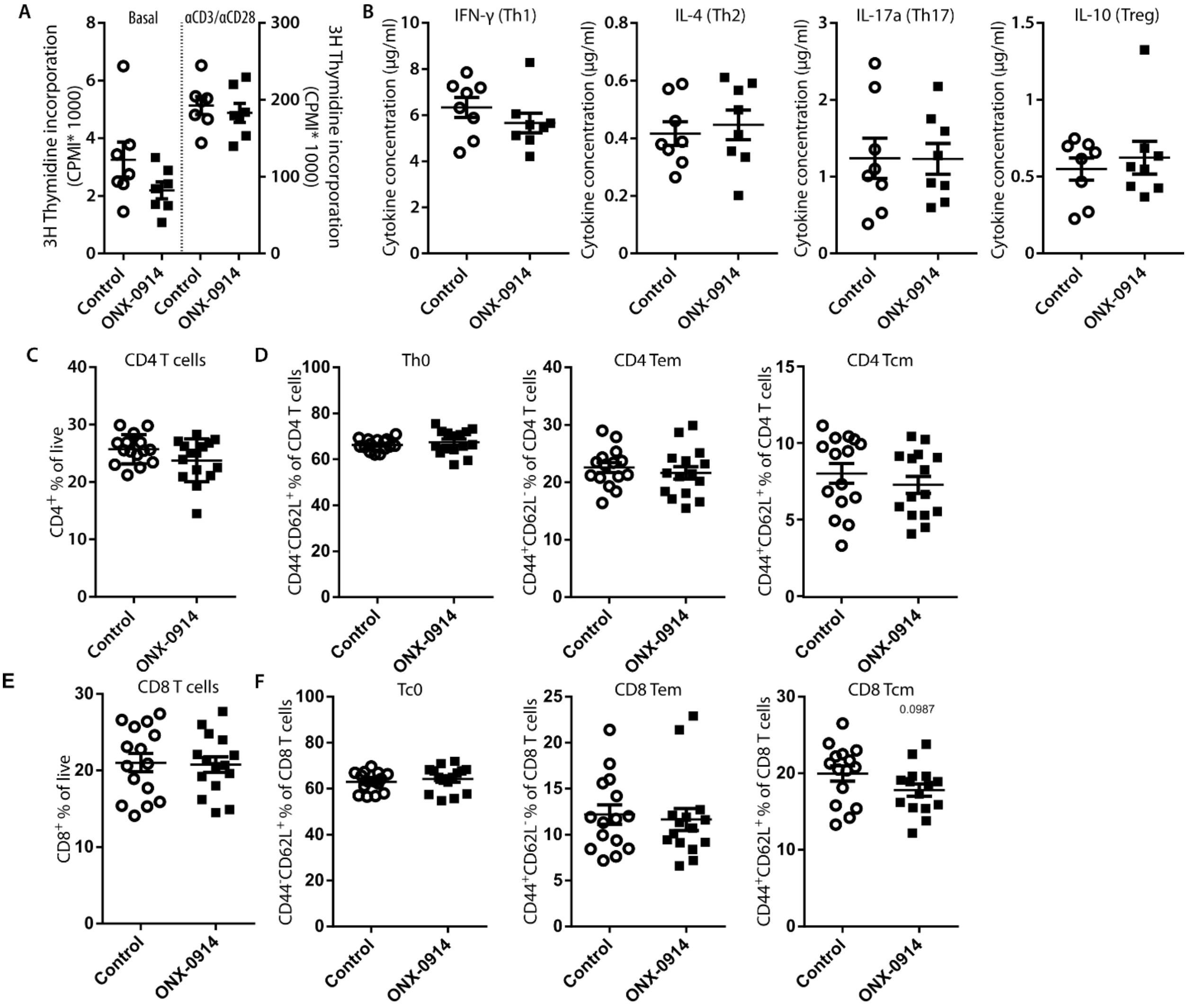

LMP7 inhibition does not impact splenic T-cell differentiation in early atherosclerosis. (A) T-cell proliferation as measured by thymidine incorporation of unstimulated or αCD3/αCD28 stimulated splenocyte cultures (n=7) derived from female LDLr$^{-/-}$ mice treated with ONX-0914 (10 mg/kg, 3 times weekly intraperitoneally) or control treated for 7 weeks, while fed a WTD. (B) Cytokine levels in supernatant of the splenocyte cultures stimulated with αCD3/αCD28 overnight. (C) Overall CD4^{+} T-cell levels, (D) CD4^{+} Tem, Tcm and Th0 levels, and (E) overall CD8^{+} T-cell levels, and (F) CD8^{+} Tem, Tcm and Tc0 levels, in the spleen as determined by flow cytometry. Expressed as mean ± SEM, unpaired two tailed t-test.

Supplemental Figure S6

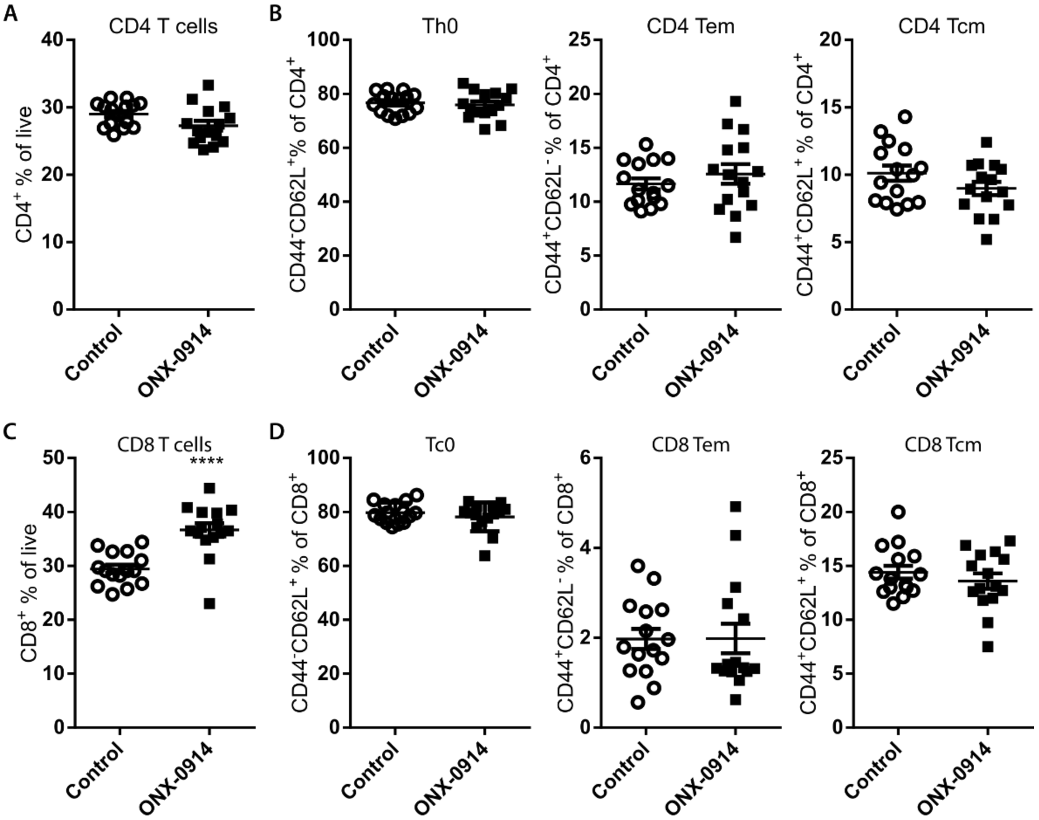

Memory and naïve T-cell effector populations in cervical lymph nodes. Quantification of flow cytometric analysis of (A) CD4+ T-cell content and (B) memory and naïve CD4+ T-cell populations, (C) CD8+ T-cells and (D) memory and naïve CD8+ T-cell populations in CLN of female LDLr–/– mice (n=15) fed a WTD for 7 weeks, during which mice were treated with ONX-0914 (10 mg/kg, 3 times weekly intraperitoneally) or control treated. Expressed as mean ± SEM, unpaired two tailed t-test, ****$p < 0.0001$.

.

Supplemental Figure S7

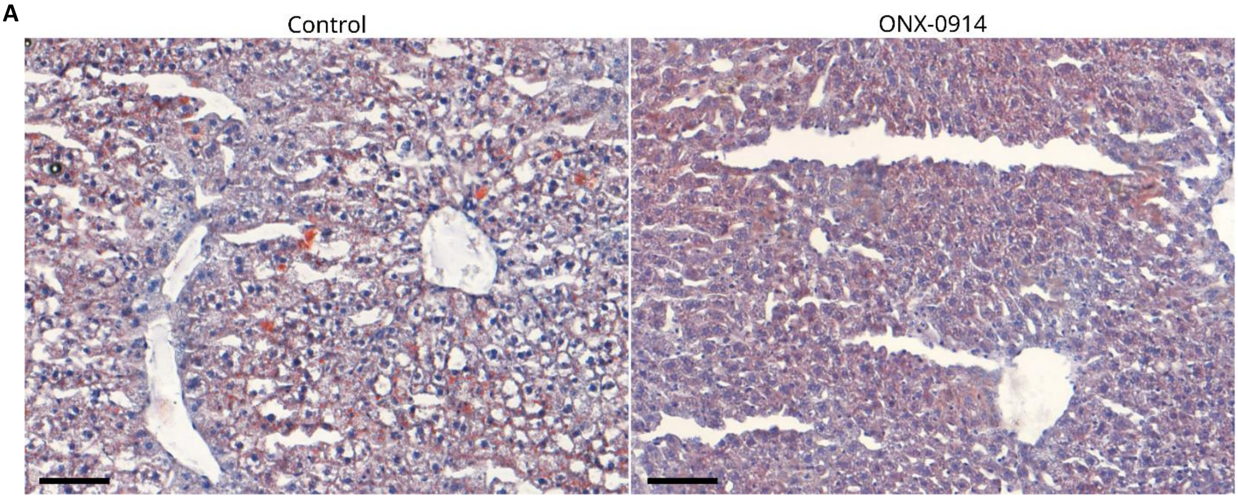

Hepatic histology. (A) Male LDLr$^{-/-}$ mice (n=8) were fed a WTD for 4 weeks after which a baseline group was sacrificed. Remaining mice were treated for 7 weeks with ONX-0914 (10 mg/kg, 3 times weekly intraperitoneally) or control treated (4% DMSO in PBS). Representative micrographs of liver sections from control and ONX-0914 treatment groups stained with Oil Red O (scale bar is 100 µm).

Supplemental Figure S8

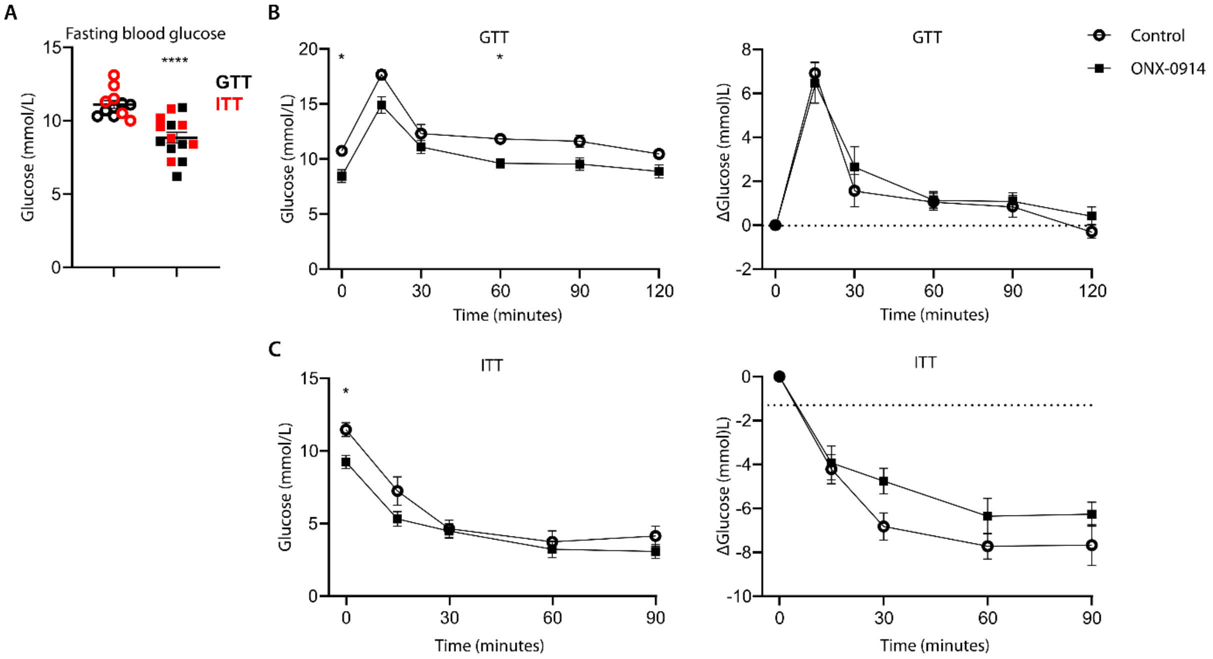

Glucose and insulin tolerance tests in WTD fed male LDLr$^{-/-}$ mice. Male LDLr$^{-/-}$ mice (n=6-7) were fed a WTD for 7 weeks after which mice were treated with ONX-0914 (10 mg/kg intraperitoneally, 3 times weekly) or control for 3 weeks while remaining on a WTD. (A) Thereafter, mice were fasted for 6h before blood glucose was assessed. Then mice (n=7 per group) (B) received an oral glucose bolus (0.06 g, 2 mg/kg for a mouse of 30 g), and blood glucose was measured 15, 30, 60, 90, and 120 minutes later, (C) or received an intraperitoneal insulin injection (0.03 Units, or 1U/kg for a 30 g mouse) and blood glucose was measured 15, 30, 60, and 90 minutes later. Expressed as mean ± SEM, * $p < 0.05$, a: Student's T-test, b,c: 2-way repeated measures ANOVA with Šídák's posthoc test.

Supplemental figure S9

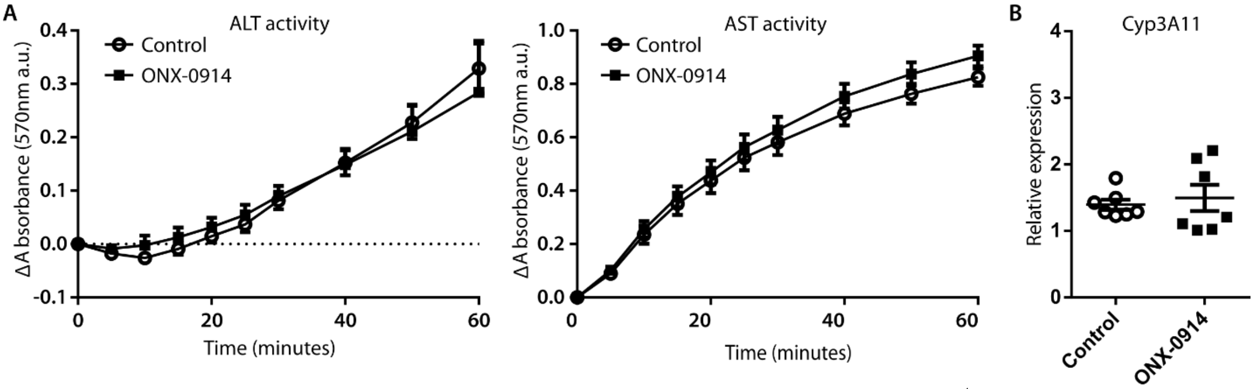

Treatment with ONX-0914 does not lead to hepatotoxicity. Male LDLr$^{-/-}$ mice (n=8) were fed a WTD after which a baseline group was sacrificed. Thereafter, mice were treated for 7 weeks with ONX-0914 (10 mg/kg, 3 times weekly intraperitoneally) or control treated (4% DMSO in PBS). (A) ALT and AST activity were assessed on blood plasma derived from blood collected at sacrifice after 7 weeks of control or ONX-0914 treatment. (B) Hepatic Cyp3A11 expression. Expressed as mean ± SEM, (A) 2-way repeated measures ANOVA with Sidak posttest, (B) two tailed t-test, no significant differences.

Supplemental figure S10

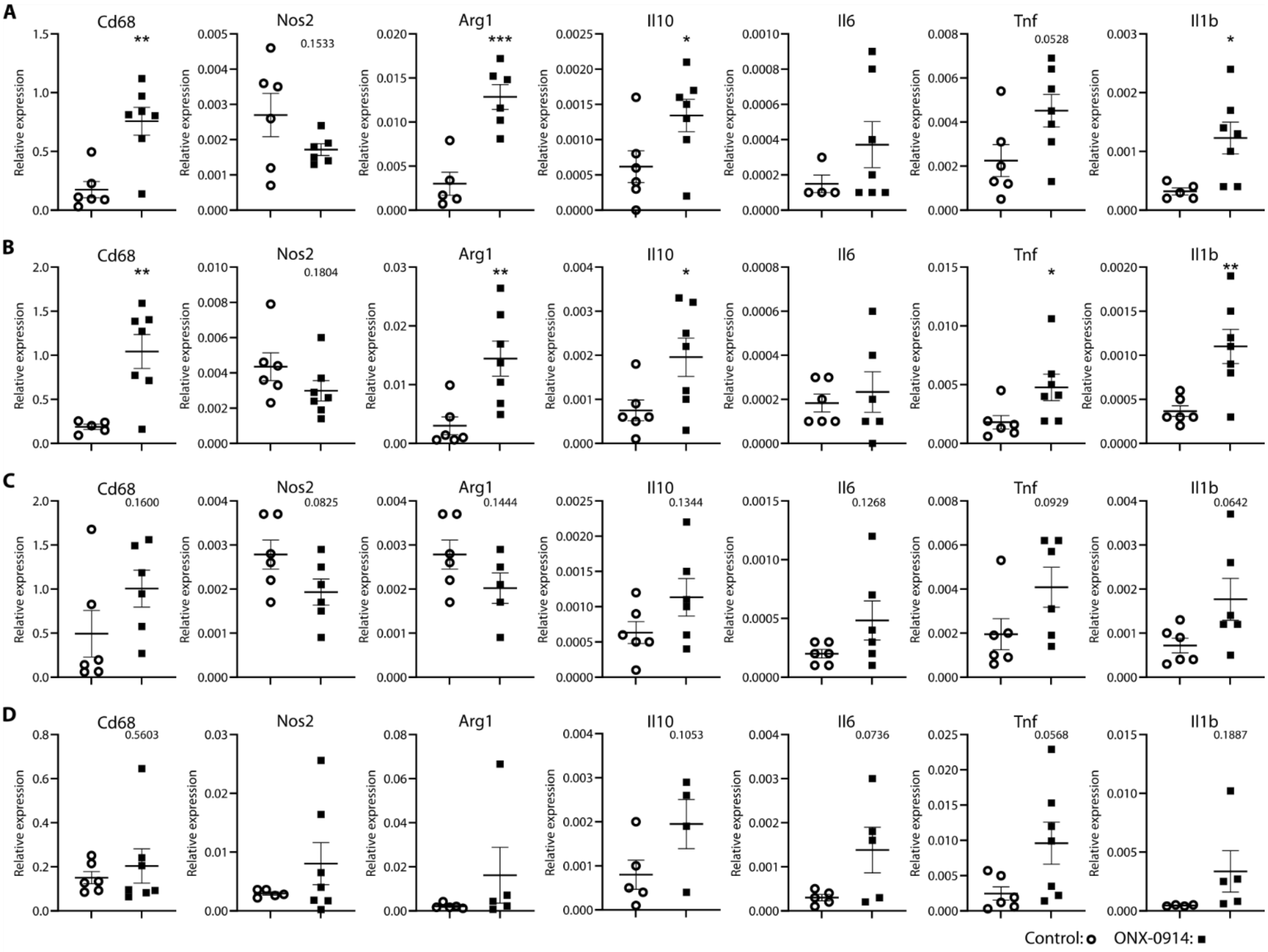

Macrophage presence in visceral white adipose tissue depots. Gene expression of macrophage related markers in (A) perigonadal, (B) retroperitoneal, (C) perirenal, and (D) mesenteric white adipose tissue depots, derived from male LDLr$^{-/-}$ mice (n=6-7) fed a WTD for 7 weeks before treatment with ONX-0914 (10 mg/kg intraperitoneally, 3 times weekly) or control for 3 weeks while on a WTD. Expressed as mean ± SEM, * $p < 0.05$, ** $p < 0.01$, *** $p < 0.001$, a-d: Student's T-tests.

Supplemental figure S11

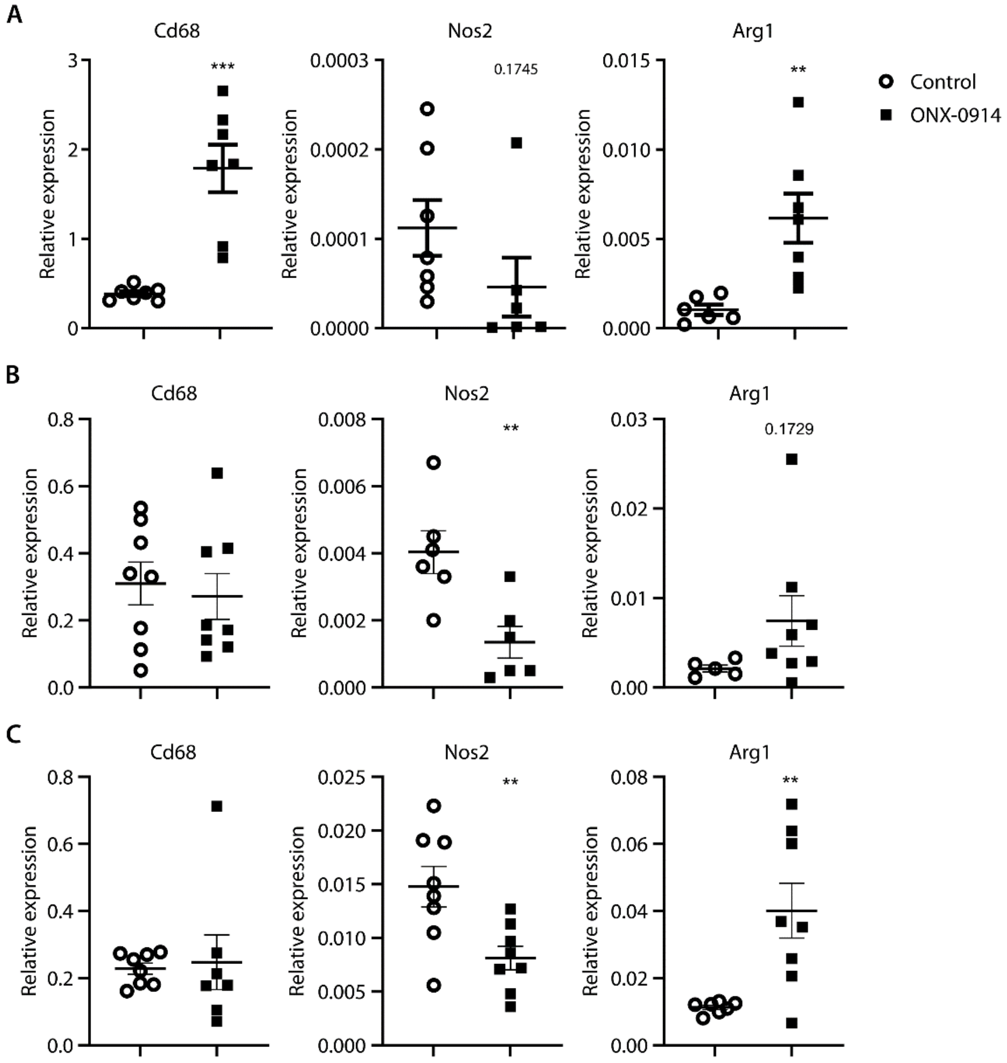

Macrophage presence in white adipose depots in female APOE*3-Leiden.CETP mice. qPCR data from (A) gonadal white adipose tissue, (B) mesenteric white adipose tissue, (C) subcutaneous adipose tissue derived from female APOE*3-Leiden.CETP mice (n=8) fed a WTD for 3 weeks after which they were treated with ONX-0914 (10 mg/kg, 3 times weekly, intraperitoneally) or control treated (4% DMSO in PBS) for 2.5 weeks.

Supplemental figure S12

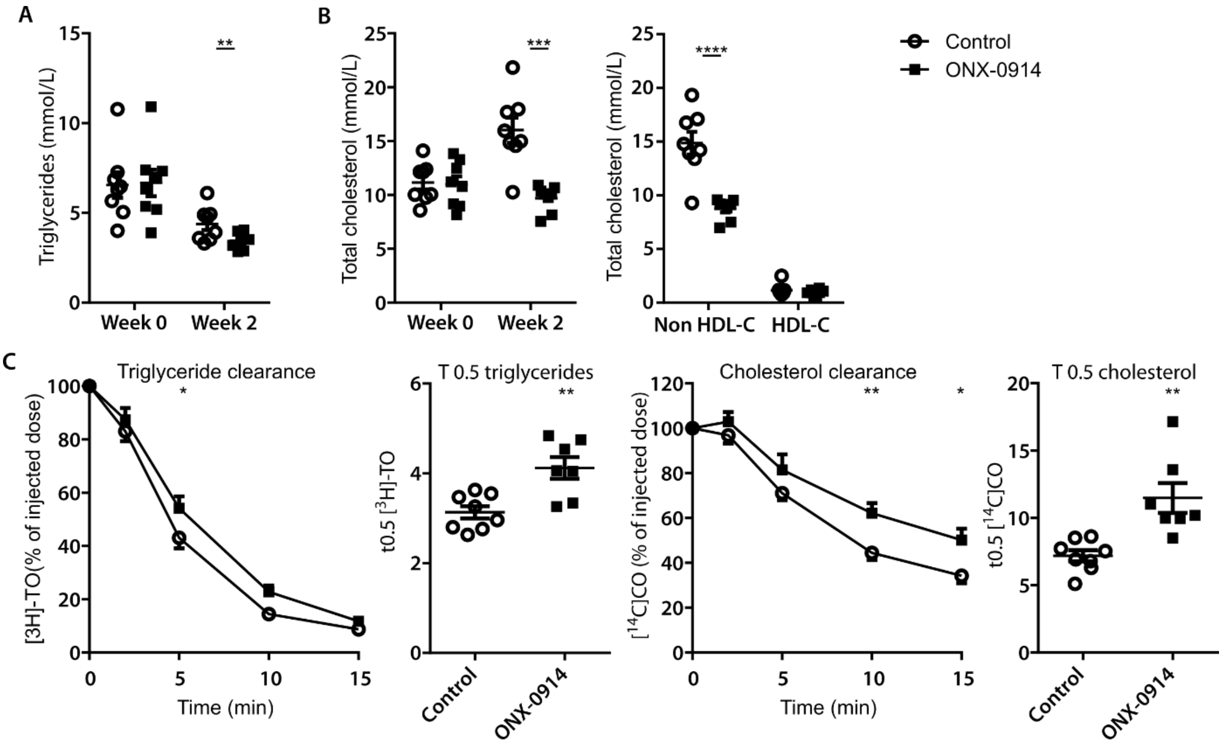

TG and TC clearance from the blood is not enhanced by ONX-0914 treatment. Female APOE*3-Leiden.CETP mice (n=8) were fed a WTD for 3 weeks after which they were treated with ONX-0914 (10 mg/kg, 3 times weekly, intraperitoneally) or control treated (4% DMSO in PBS) for 2.5 weeks. After 2.5 weeks mice received a final control or ONX-0914 injection and were fasted for 4h. (A) Plasma triglyceride levels and (B) cholesterol levels in plasma after 4h of fasting. (C) To determine clearance of TG and TC from the blood, VLDL like particles containing radiolabeled [^{14}C]cholesteryl oleate and glycerol tri[^{3}H]oleate were administered intravenously. Radioactivity in plasma was assessed in blood drawn at indicated time points after VLDL like particle administration, and half life time was approximated. Expressed as mean ± SEM (A,B) (left panel), C (1st and 3rd panel from the right), two tailed T-test, (B) (right panel) one-way ANOVA with Sidak posttest, C (1st and 3rd panel from the left) two-way repeated measures ANOVA with Sidak posttest, * $p < 0.05$, ** $p < 0.01$, *** $p < 0.001$, **** $p < 0.0001$

Supplemental Figure S13

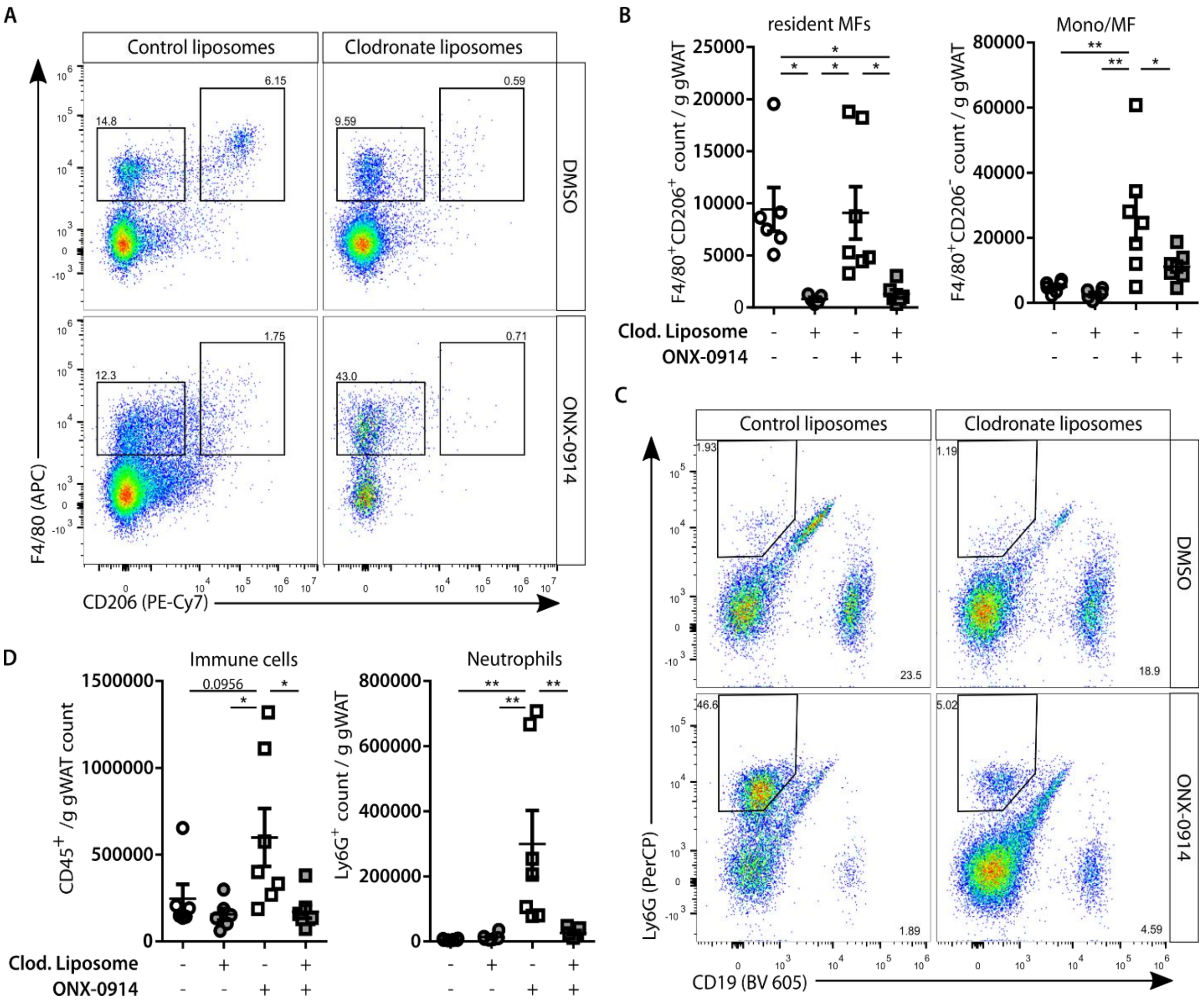

Depletion of peritoneal macrophages in gWAT. Female LDLr$^{-/-}$ mice (n=8) were fed a WTD for 27 weeks after which they were treated with ONX-0914 (10 mg/kg, 3 times weekly, intraperitoneally) or control treated (4% DMSO in PBS) for 1 week. A day prior to ONX-0914 treatment, half of the mice received clodronate liposomes or control liposomes. SVF from gWAT was isolated for flow cytometric analysis. (A) Representative flow cytometry plot of live single CD45$^+$ cells gated for F480$^+$CD206$^+$ resident macrophages and F480$^+$CD206$^-$ infiltrating macrophages (B) Quantification of resident and infiltrating macrophage levels. (C) Flow cytometric gating of Ly6G$^+$ neutrophils in SVF, and (D) quantification of overall CD45$^+$ immune cells and neutrophils in SVF of gWAT. Expressed as mean ± SEM, One-way ANOVA with Sidak posttest, * $p < 0.05$, ** $p < 0.01$.

Supplemental Figure S14

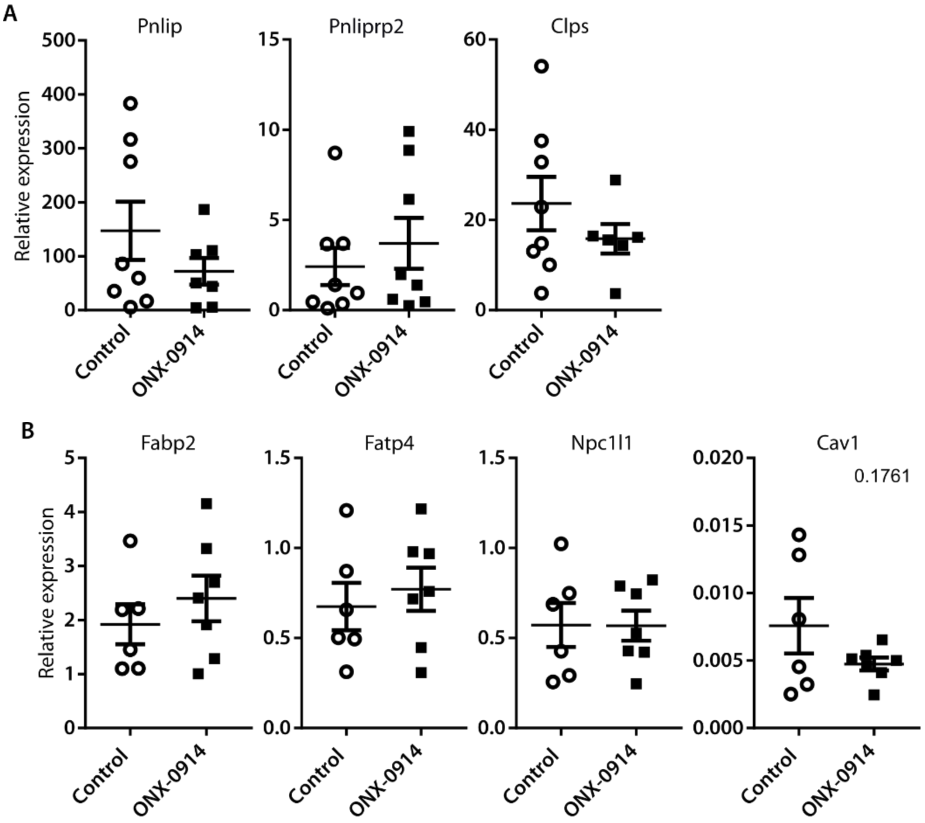

ONX-0914 does not affect expression of pancreatic lipases or receptors involved in intestinal lipid uptake. (A) Expression of pancreatic lipase (Pnlip), pancreatic lipase-related protein 2-like (Pnliprp2), and colipase (Clps) in pancreatic tissue isolated from female E3L.CETP mice on WTD for 5.5 weeks, treated with ONX-0914 for the final 2.5 weeks (10 mg/kg, 3 times weekly). (B) Gene expression of the lipid transporters fatty acid binding protein-2 (Fabp2), fatty acid transfer protein 4 (Fatp4), caveolin 1 (Cav1), and NPC1-like intracellular cholesterol transporter 1 (Npc1l1), in intestines from female LDLr$^{-/-}$ females on WTD for 6 months, ip treated with ONX-0914 (10 mg/kg, 3 times weekly) in the final week. Expressed as mean ± SEM, unpaired two-tailed t-test, no significant data.

Supplemental Figure S15

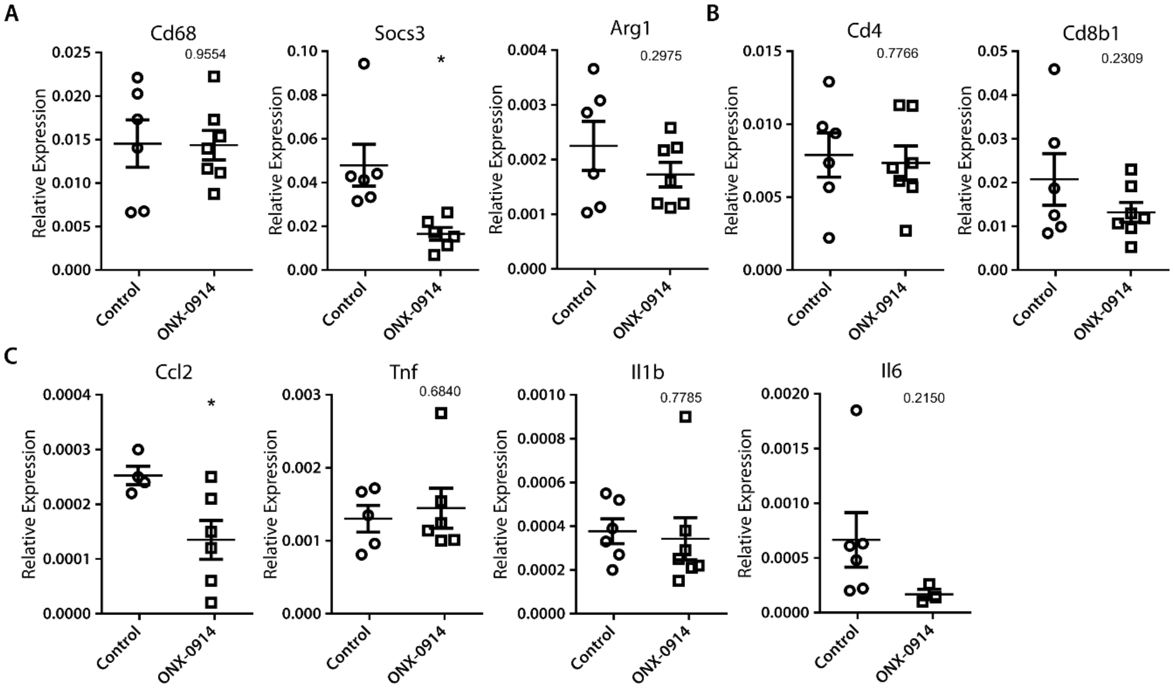

Immunoproteasomal inhibition does not cause intestinal inflammation. Small intestines of LDLr$^{-/-}$ mice fed a WTD for 28 weeks and treated with ONX-0914 or control for the final week, were assessed for (A) macrophage, (B) T cell, and (C) inflammatory cytokine content by gene expression analysis. Expressed as mean ± SEM, unpaired two tailed t-test, * p < 0.05.

Supplemental Figure S16

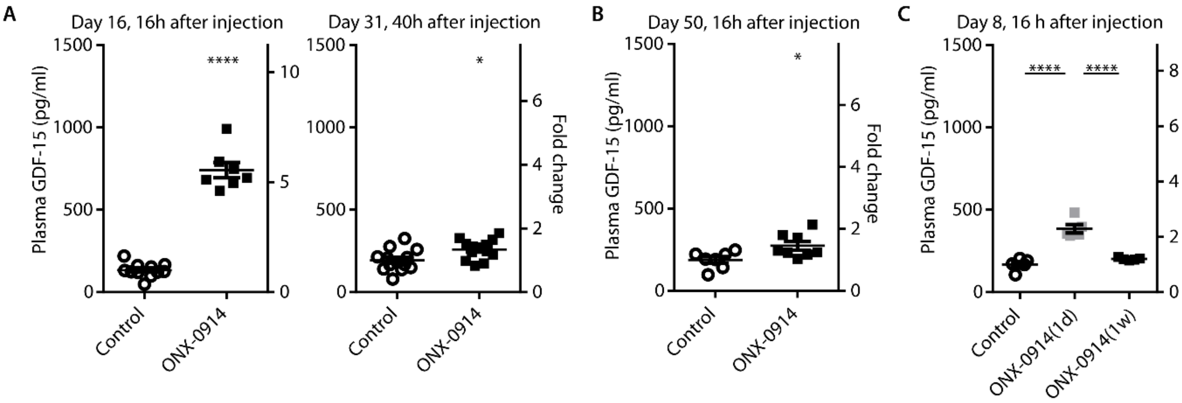

ONX-0914 induces GDF15. GDF15 levels in blood plasma of (A) female LDLr$^{-/-}$ mice (n=15) after treatment with ONX-0914 (10 mg/kg, 3 times weekly intraperitoneally) or control treated for 7 weeks, while fed a WTD, (B) of male LDLr$^{-/-}$ mice (n=8) fed a WTD for 4 weeks after which mice were treated for 7 weeks with ONX-0914 (10 mg/kg, 3 times weekly intraperitoneally) or control treated while remaining on a WTD, (C) of female LDLr$^{-/-}$ mice (n=5-6) fed a western type diet for 6 weeks, followed by treatment for 1 week (4 ONX-0914 injections) or 1 day (3 control injections followed by 1 ONX-0914 injection), or with vehicle injections (4 control injections). Expressed as mean ± SEM, unpaired two tailed t-test, * p < 0.05, **** p < 0.0001.

Gating strategies

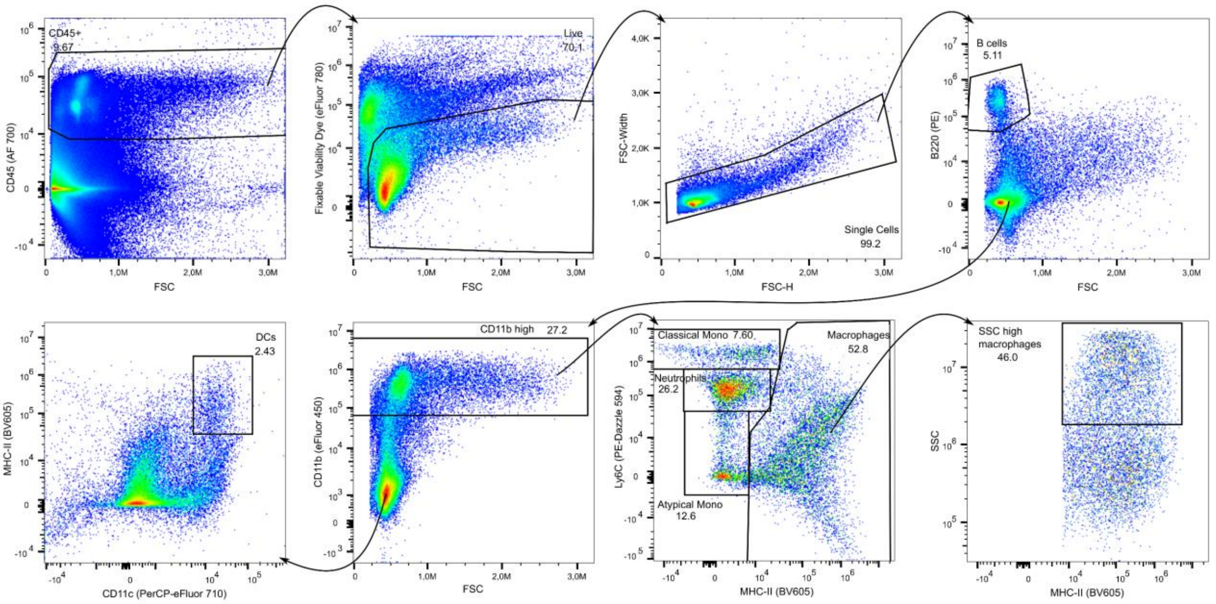

Gating strategy for various immune populations in the aortic arch (Figure 2A-D). CD45$^+$ cells were gated, after which fixable viability dye low cells were gated to remove dead cells, and singlets were gated (used for cell count). Then, B cells (B220$^+$) cells were gated. From the B220$^-$ population CD11b^{high} cells were gated from which classical monocytes (CD11b^{high}Ly6C^{high}MHC-II$^-$), neutrophils (CD11b^{high}Ly6C^{mid}MHC-II$^-$), atypical monocytes (CD11b^{high}Ly6C^{low}MHC-II$^-$), and macrophages (CD11b^{high}MHC-II$^+$) were gated. The macrophage population was then subdivided in a SSC^{high} and SSC^{low} population. From the B220$^-$CD11b^{low} population dendritic cells were gated (MHC-II$^+$CD11c$^+$).

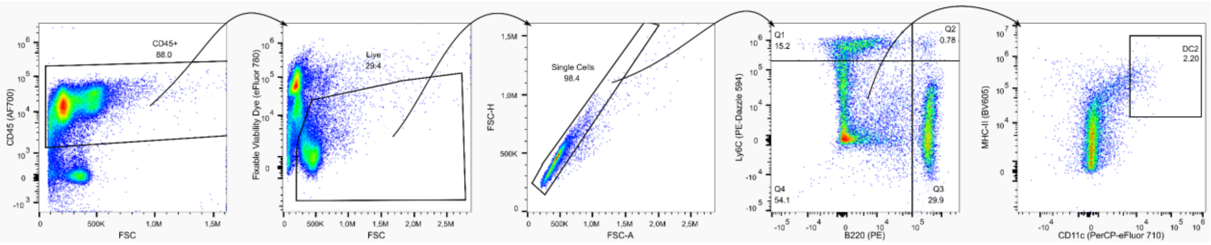

Gating strategy for dendritic cells in mediastinal (heart) lymph nodes (Figure 2E/F). CD45$^+$ cells were gated, after which fixable viability dye low cells were gated to remove dead cells, and singlets were gated (used for cell count). Then Ly6C^{low}B220^{low} cells were gated to gate the MCH-II$^+$CD11c$^+$ dendritic cells.

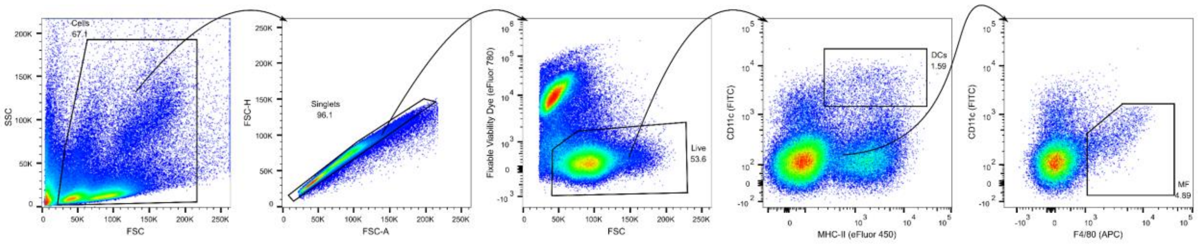

Gating strategy for dendritic cells and macrophages (Figure 3). Cells were gated based on FSC-SSC, after which doublets were removed and fixable viability dye low cells were gated. Then MCH-II^{+}CD11c^{+} dendritic cells were gated, and subsequently F4/80^{+} red pulp macrophages were gated from the non-DC cell population in spleen samples. Gating shown for a representative spleen sample.

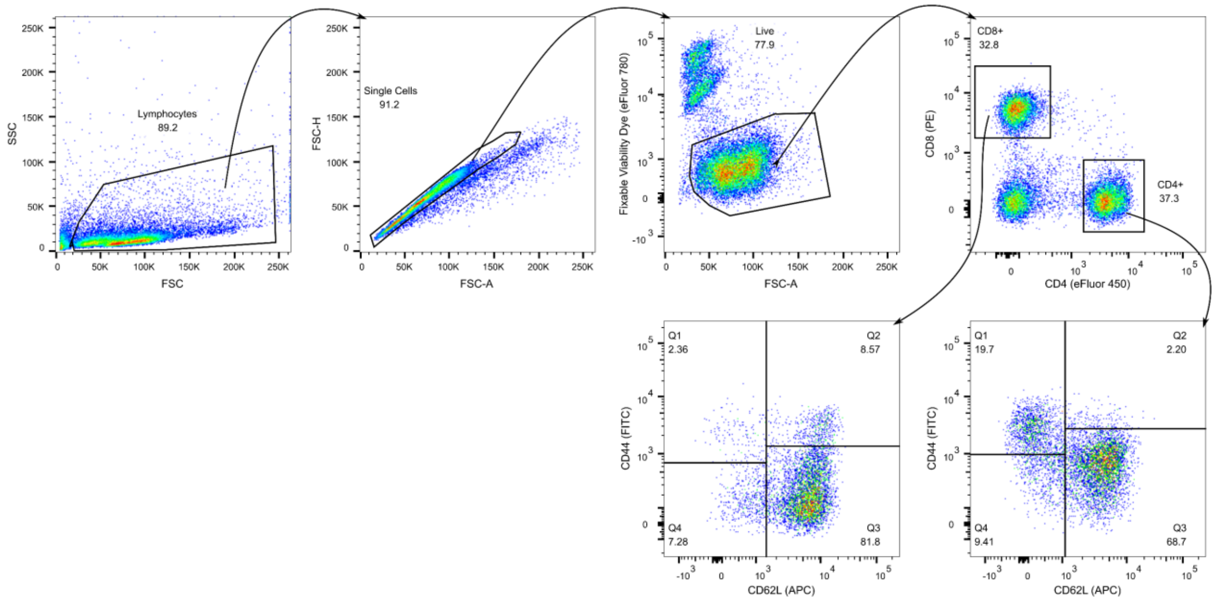

Gating of CD4^{+} and CD8^{+} memory populations (Figure 4A-D, Supplemental Figure S5C-F and S6A-D). Cells were gated based on FSC and FSC. Singlets were gated, followed by exclusion of dead cells (Fixable Viability Dye^{low}). Then CD4^{+} and CD8^{+} cells were gated and divided in naïve (CD44^{-}CD62L^{+}), effector memory (CD44^{+}CD62L^{-}) and central memory (CD44^{+}CD62L^{-}) populations. Gating shown for a representative mesenteric lymph node sample.

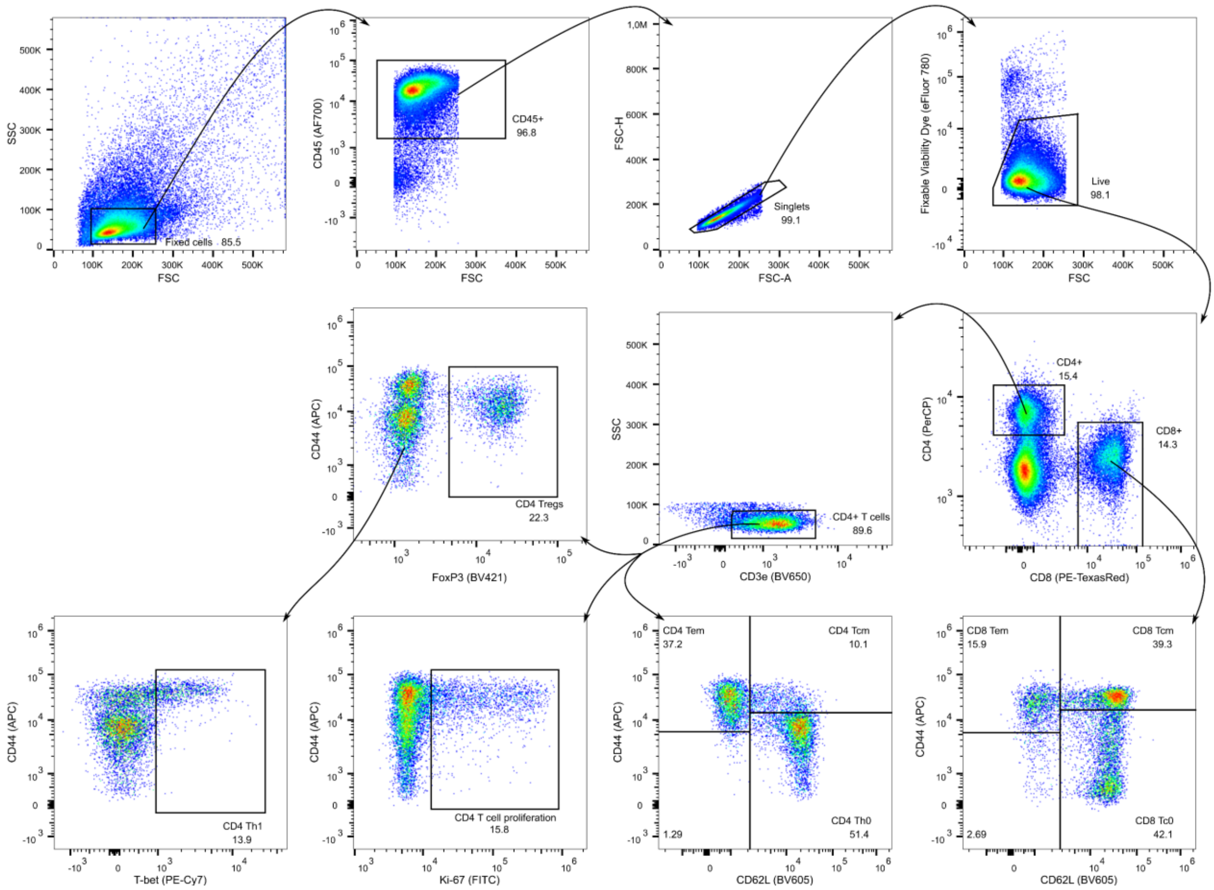

Gating of T cell analysis (Figure 4E-F, Supplemental Figure S4B/C). Fixed cells were gated based on FSC vs SSC. Subsequently, leukocytes ($CD45^+$) and singlets (FSC-A vs FSC-H), whereafter live cells were gated (Fixable Viability Dye^{low}). Thereafter, $CD4^+$ and $CD8^+$ cells were gated. From the $CD4^+$ cells, $CD3^+$ cells were selected, from which $FoxP3^+CD4^+$ Tregs, proliferating $CD4^+$ T cells ($Ki\text{-}67^+$), naïve ($CD62L^+CD44^-$) effector memory ($CD62L^-CD44^+$) and central memory ($CD62L^+CD44^+$) populations were gated. From the $CD4^+FoxP3^-$ population Th1 ($T\text{-}bet^+$) cells were gated. Similar gatings were performed for the $CD8^+$ T cell population as for the $CD4^+$ population but these data were not used in this manuscript. Gatings shown for a representative spleen sample.

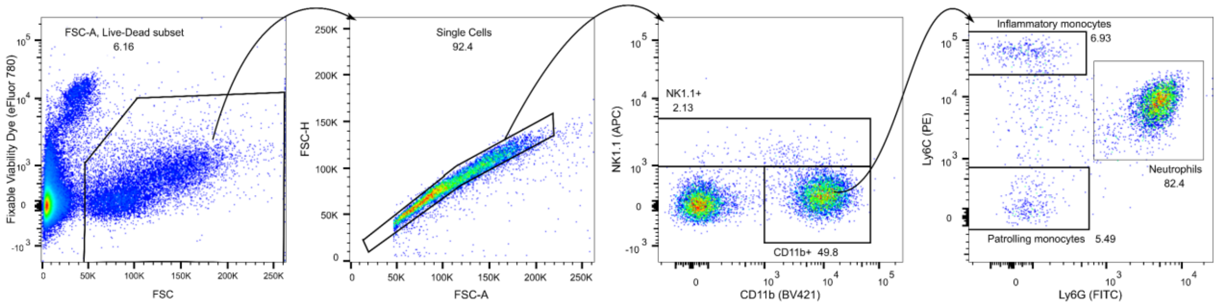

Gating of myeloid cell populations in blood (Figure 5B). Live cells were gated based on FSC and fixable viability dye intensity. Singlets were gated after which $CD11b^+NK1.1^-$ cells were gated and subdivided in a Neutrophil gate ($Ly6C^{mid}Ly6G^+$), inflammatory monocyte gate ($Ly6C^{high}Ly6G^-$) and a patrolling monocyte gate ($Ly6C^-Ly6G^-$). Gating shown for a representative blood sample from the ONX-0914 treated group.

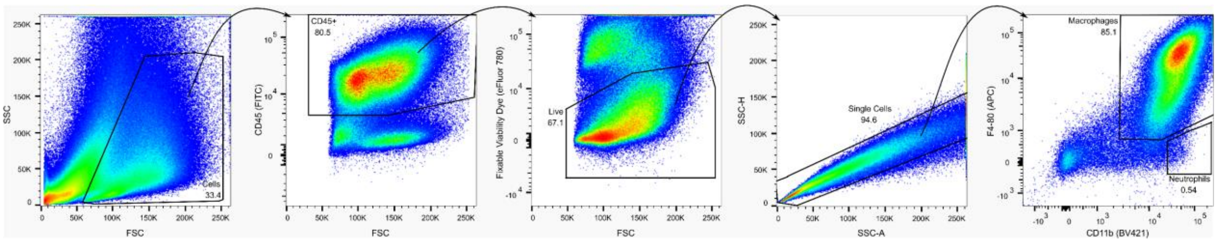

Gating of myeloid cell populations in SVF from gWAT (Figure 6A). Cells were gated based on FSC SSC. From there, leukocytes were gated based on CD45⁺ expression. Thereafter live cells were gated (Fixable Viability Dye^{low}), and singlets were gated based on SSC-A and SSC-H. Gating shown on a representative SVF sample from the ONX-0914 treated group.

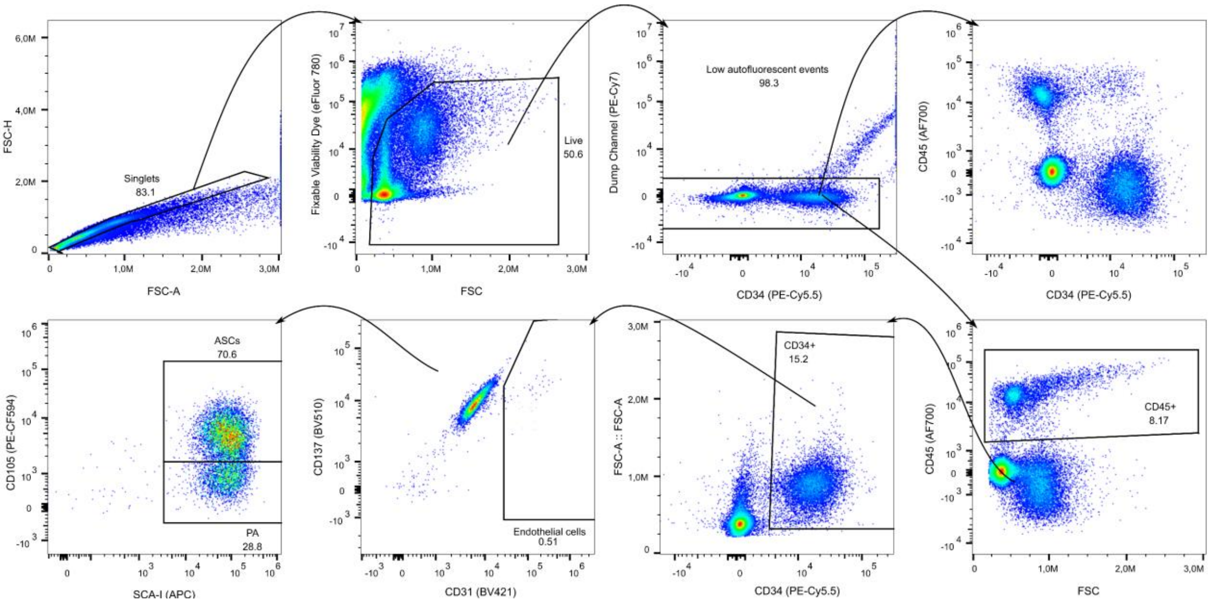

Gating of adipocyte progenitor populations in SVF from gWAT (Figure 6B). Singlets were gated based on FSC-H vs FSC-A. Subsequently, live cells were gated (Fixable Viability Dye^{low}). Thereafter high-autofluorescent cells were gated out using an empty channel, leading to the plot depicted in Figure 6B, leukocytes were gated out based on CD45⁺ expression, and CD34⁺ cells were then gated. CD31⁺ cells were gated out to remove endothelial cells from the CD34⁺ population. Then adipose stem cells (SCA-I⁺CD105⁺) and preadipocytes were gated (SCA-I⁺CD105⁻). Gating shown for a SVF sample from the control treated group.

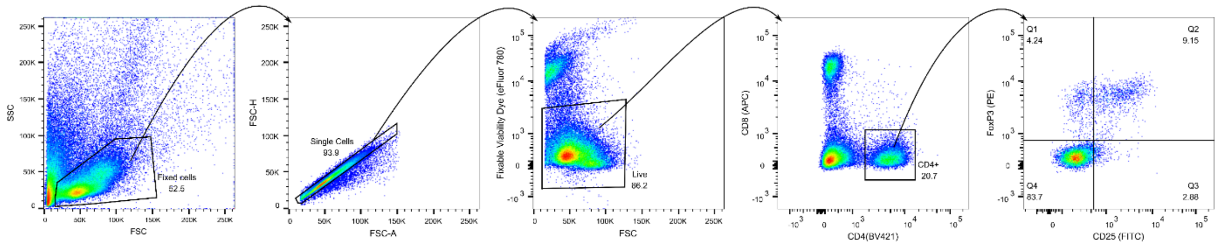

Gating of Tregs (Supplemental Figure S4A). Fixed cells were gated based on FSC vs SSC. Subsequently, single cells were gated based on FSC-A and FSC-H, whereafter live cells were gated (Fixable Viability Dye^{low}). CD4⁺ cells were gated, after which FoxP3⁺CD25⁺ and FoxP3⁺CD25⁻ CD4⁺ Treg populations were gated. Gating shown for a representative spleen sample.

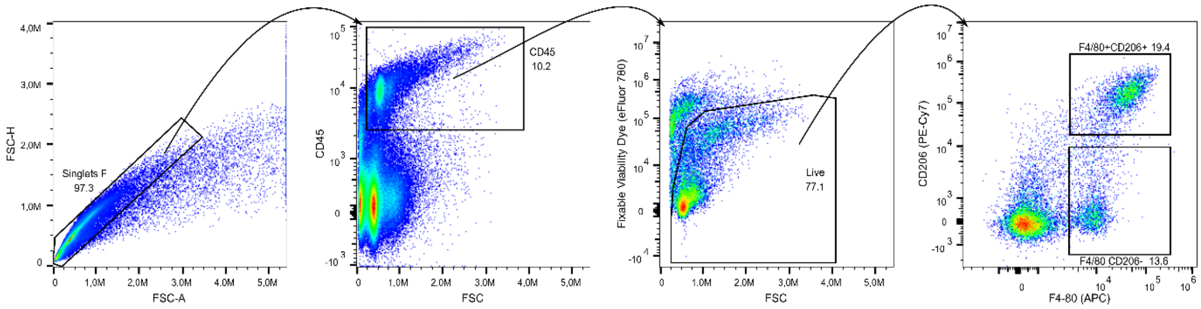

Gating of Macrophage populations in SVF (Supplemental Figure S10A). Singlets were gated after which CD45⁺ cells were selected. From there, live cells were gated and the F4/80⁺CD206⁻ and F4/80⁺CD206⁺ macrophage populations were gated.

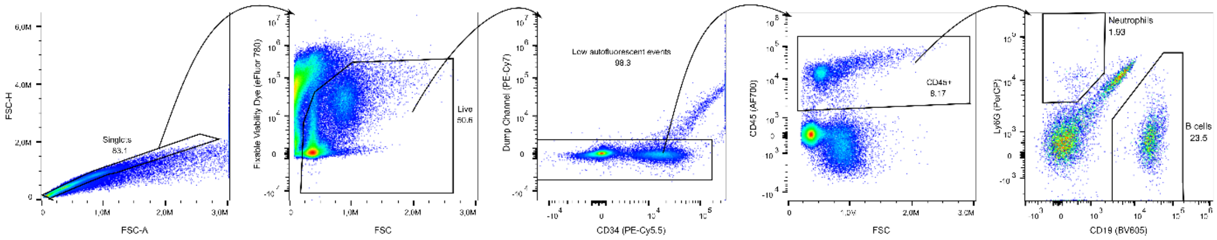

Gating of Neutrophils in SVF (Supplemental Figure S10C). Singlets were gated based on FSC-A and FSC-H. Thereafter, live cells (Fixable Viability Dye^{low}), low autofluorescent cells, and CD45⁺ cells were gated to gate the live single leukocytes. From there, Neutrophils were gated (Ly6G⁺). Gating shown for a representative SVF sample from the control treated group.

Major Resources Table

Animals (in vivo studies)

Species	Vendor or Source	Background Strain	Sex	Persistent ID / URL
Mus musculus	Jackson/own breeding	C57BL/6 Ldlr^{tm1Her}	Female/Male	https://www.jax.org/strain/002207
Mus musculus	Leiden University Medical Center	C57BL/6J APOE*3-Leiden.CETP	Female	C57BL/6J APOE*3-Leiden.CETP

Antibodies

Target antigen (conjugate)	Vendor or Source	Catalog #	Dilution / Working concentration	Lot #	Persistent ID (clone) / URL
MOMA-2	Sanbio	MCA519G	1:1000 (0.5 μmg/mL)		MOMA-2, https://www.sanbio.nl/catalog/product/view/id/680458
Goat anti-rat Ig	Sigma Aldrich	A8438	1:1000		A8438, https://www.sigmaaldrich.com/NL/en/product/sigma/a8438
αCD16/32	Biolegend	101302	1:250 (2 μg/mL)		93, https://www.biolegend.com/en-us/products/purified-anti-mouse-cd16-32-antibody-190
CD4 (PerCP)	BD Pharmingen	553049	1:1600 (0. 125 μg/mL)		RM4-5, https://www.bdbiosciences.com/en-nl/products/reagents/flow-cytometry-reagents/research-reagents/single-color-antibodies-ruo/pe-rat-anti-mouse-cd4.553049
CD4 (eFluor 450)	Thermofisher (eBioscience)	48-0041-82	1:500		GK1.5, https://www.thermofisher.com/antibody/product/CD4-Antibody-clone-GK1-5-Monoclonal/48-0041-82
CD105 (PE-CF594)	BD Pharmingen	562759	1:1600 (0. 125 μg/mL)		MJ7/18, https://www.bdbiosciences.com/en-nl/products/reagents/flow-cytometry-reagents/research-reagents/single-color-antibodies-ruo/pe-rat-anti-mouse-cd105.562759

CD31 (BV421)	BD Pharmingen	562939	1:1600 (0. 125 µg/mL)		MEC 13.3, https://www.bdbiosciences.com/en-nl/products/reagents/flow-cytometry-reagents/research-reagents/single-color-antibodies-ruo/bv421-rat-anti-mouse-cd31.562939
CD45 (FITC)	BD Pharmingen	553080	1:900 (0.556 µg/mL)		30-F11, https://www.bdbiosciences.com/en-nl/products/reagents/flow-cytometry-reagents/research-reagents/single-color-antibodies-ruo/fitc-rat-anti-mouse-cd45.553080
Ly6G (FITC)	BD Pharmingen	551460	1:1800 (0.278 µg/mL)		1A8, https://www.bdbiosciences.com/en-nl/products/reagents/flow-cytometry-reagents/research-reagents/single-color-antibodies-ruo/fitc-rat-anti-mouse-ly-6g.551460
CD11b (BV421)	Biolegend	101236	1:1800 (0.111 µg/mL)		M1/70, https://www.biolegend.com/en-us/products/brilliant-violet-421-anti-mouse-human-cd11b-antibody-7163
CD11c (FITC)	Biolegend	117306	1:900 (0.556 µg/mL)		N418, https://www.biolegend.com/en-us/products/fitc-anti-mouse-cd11c-antibody-1815
CD44 (BV421)	Biolegend	103040	1:1000 (0.2 µg/mL)		IM7, https://www.biolegend.com/en-us/products/brilliant-violet-421-anti-mouse-human-cd44-antibody-7225
CD44 (FITC)	Biolegend	103006	1:900 (0.556 µg/mL)		IM7, https://www.biolegend.com/en-us/products/fitc-anti-mouse-human-cd44-antibody-314
CD45 (AF700)	Biolegend	103128	1:3200 (0.156 µg/mL) 0.5 mg/mL		30-F11, https://www.biolegend.com/en-us/products/alexa-fluor-700-anti-mouse-cd45-antibody-3407
CD8 (BV510)	Biolegend	100752	1:900 (0.222 µg/mL)		53-6.7, https://www.biolegend.com/en-us/products/brilliant-violet-510-anti-mouse-cd8a-antibody-7992
CD8 (APC)	Biolegend	100712	1:900 (0.222 µg/mL)		53-6.7, https://www.biolegend.com/en-us/products/apc-anti-mouse-cd8a-antibody-150
CD206 (PE-Cy7)	Biolegend	141720	1:1600 (0.125 µg/mL)		C068C2, https://www.biolegend.com/en-us/products/pe-cyanine7-anti-mouse-cd206-mmr-antibody-8631
CD34 (PE-Cy5)	Biolegend	119312	1:3200 (0.0625 µg/mL)		MEC14.7, https://www.biolegend.com/en-us/products/pe-cyanine5-anti-mouse-cd34-antibody-3943

CD115 (PE)	eBioscience/ Thermofisher	12-1152-83	1:1800 (0.111 µg/mL)		AFS98, https://www.thermofisher.com/antibody/product/CD115-c-fms-Antibody-clone-AFS98-Monoclonal/12-1152-83
CD11c (PE)	eBioscience/ Thermofisher	12-0114-82	1:900 (0.222 µg/mL)		N418, https://www.thermofisher.com/antibody/product/CD11c-Antibody-clone-N418-Monoclonal/12-0114-82
CD19 (BV421)	eBioscience/ Thermofisher	404-0193-82	1:900 (0.222 µg/mL)		eBio1D3, https://www.thermofisher.com/antibody/product/CD19-Antibody-clone-eBio1D3-1D3-Monoclonal/404-0193-82
CD19 (PE-Cy7)	eBioscience/ Thermofisher	25-0193-82	1:1800 (0.111 µg/mL)		eBio1D3, https://www.thermofisher.com/antibody/product/CD19-Antibody-clone-eBio1D3-1D3-Monoclonal/25-0193-82
CD25 (APC)	eBioscience/ Thermofisher	17-0251-82	1:900 (0.222 µg/mL)		PC61.5, https://www.thermofisher.com/antibody/product/CD25-Antibody-clone-PC61-5-Monoclonal/17-0251-82
CD25 (FITC)	eBioscience/ Thermofisher	11-0251-82	1:450 (1.11 µg/mL)		PC61.5, https://www.thermofisher.com/antibody/product/CD25-Antibody-clone-PC61-5-Monoclonal/11-0251-82
CD3e (PE-Cy5)	eBioscience/ Thermofisher	15-0031-82	1:6400 (0.0313 µg/mL)		145-2C11, https://www.thermofisher.com/antibody/product/CD3e-Antibody-clone-145-2C11-Monoclonal/15-0031-82
CD34 (eFluor 450)	eBioscience/ Thermofisher	48-0341-82	1:900 (0.222 µg/mL)		RAM34, https://www.thermofisher.com/antibody/product/CD34-Antibody-clone-RAM34-Monoclonal/48-0341-82
CD44 (APC)	eBioscience/ Thermofisher	17-0441-82	1:900 (0.222 µg/mL)		IM7, https://www.thermofisher.com/antibody/product/CD44-Antibody-clone-IM7-Monoclonal/17-0441-82
CD62L (APC)	eBioscience/ Thermofisher	17-0621-82	1:1800 (0.111 µg/mL)		MEL-14, https://www.thermofisher.com/antibody/product/CD62L-L-Selectin-Antibody-clone-MEL-14-Monoclonal/17-0621-82
CD62L (Pacific Blue)	eBioscience/ Thermofisher	RM4328	1:900 (0.111 µg/mL)		MEL-14, https://www.thermofisher.com/antibody/product/CD62L-Antibody-clone-MEL-14-Monoclonal/RM4328
CD80 (PE)	eBioscience/ Thermofisher	12-0801-82	1:900 (0.111 µg/mL)		16-10A1, https://www.thermofisher.com/antibody/product/CD80-B7-1-Antibody-clone-16-10A1-Monoclonal/12-0801-82
CD86 (PE)	eBioscience/ Thermofisher	12-0862-82	1:900 (0.111 µg/mL)		GL1, https://www.thermofisher.com/antibody/product/CD86-B7-2-Antibody-clone-GL1-Monoclonal/12-0862-82
F4/80 (APC)	eBioscience/ Thermofisher	17-4801-82	1:900 (0.111 µg/mL)		BM8, https://www.thermofisher.com/antibody/product/F4-80-Antibody-clone-BM8-Monoclonal/17-4801-82

FoxP3 (Pacific Blue/ eFluor 450)	eBioscience/ Thermofisher	48-5773-82	1:1000 (0.2 µg/mL)		FJK-16s, https://www.thermofisher.com/antibody/product/FOXP3-Antibody-clone-FJK-16s-Monoclonal/48-5773-82
FoxP3 (PE)	eBioscience/ Thermofisher	12-4771-82	1:500 (0.4 µg/mL)		NRRF-30, https://www.thermofisher.com/antibody/product/FOXP3-Antibody-clone-NRRF-30-Monoclonal/12-4771-82
Ly6C (PE)	eBioscience/ Thermofisher	12-5932-82	1:3000 (0.0667 µg/mL)		HK1.4, https://www.thermofisher.com/antibody/product/Ly-6C-Antibody-clone-HK1-4-Monoclonal/12-5932-82
Ly6C (PerCP-Cy5.5)	eBioscience/ Thermofisher	45-5932-82	1:1800 (0.111 µg/mL)		HK1.4, https://www.thermofisher.com/antibody/product/Ly-6C-Antibody-clone-HK1-4-Monoclonal/45-5932-82
NK1.1 (APC)	eBioscience/ Thermofisher	17-5941-82	1:800 (0.25 µg/mL)		PK136, https://www.thermofisher.com/antibody/product/NK1-1-Antibody-clone-PK136-Monoclonal/17-5941-82
T-bet (PE-Cy7)	eBioscience/ Thermofisher	25-5825-82	1:1600 (0.125 µg/mL)		eBio4B10, https://www.thermofisher.com/antibody/product/T-bet-Antibody-clone-eBio4B10-4B10-Monoclonal/25-5825-82
CD11b (BV605)	eBioscience/ Thermofisher	406-0112-82	1:200 (1 µg/mL)		M1/70, https://www.thermofisher.com/antibody/product/CD11b-Antibody-clone-M1-70-Monoclonal/406-0112-82
CD161 (PE)	eBioscience/ Thermofisher	12-1619-42	1:3200 (0.0313 µg/mL)		HP-3G10, https://www.thermofisher.com/antibody/product/CD161-Antibody-clone-HP-3G10-Monoclonal/12-1619-42
CD86 (PE-Cy5)	eBioscience/ Thermofisher	15-0862-82	1:6400 (0.0313 µg/mL)		GL1, https://www.thermofisher.com/antibody/product/CD86-B7-2-Antibody-clone-GL1-Monoclonal/15-0862-82
CD8a (PE-TR)	eBioscience/ Thermofisher	MCD0817	1:6400 (0.0313 µg/mL)		5H10, https://www.thermofisher.com/antibody/product/CD8-alpha-Antibody-clone-5H10-Monoclonal/MCD0817
SCA-I (APC)	eBioscience/ Thermofisher	17-5981-83	1:6400 (0.0313 µg/mL)		D7, https://www.thermofisher.com/antibody/product/Ly-6A-E-Sca-1-Antibody-clone-D7-Monoclonal/17-5981-83
MHC-II (PerCP-eFluor 710)	eBioscience/ Thermofisher	46-5321-82	1:900 (0.222 µg/mL)		M5/114.15.2, https://www.thermofisher.com/antibody/product/MHC-Class-II-I-A-I-E-Antibody-clone-M5-114-15-2-Monoclonal/46-5321-82
MCH-II (eVolve 655)	eBioscience/ Thermofisher	86-5321-42	1:800		M5/114.15.2, https://www.thermofisher.com/antibody/product/MHC-Class-II-I-A-I-E-Antibody-clone-M5-114-15-2-Monoclonal/86-5321-42
SCA-I (PE)	eBioscience/ Thermofisher	12-5981-82	0.2 mg/mL ()		D7, https://www.thermofisher.com/antibody/product/Ly-6A-E-Sca-1-Antibody-clone-D7-Monoclonal/12-5981-82

Other

Description	Source	Persistent ID / URL
ONX-0914	Leiden Institute of Chemistry	https://www.medchemexpress.com/ONX-0914.html ((Alternative source)
GastroSense 750	Perkin Elmer	https://www.perkinelmer.com/nl/product/ivisense-gastrointestinal-750-nev11121
Clodronate liposomes	Liposoma	https://clodronateliposomes.com/product/clodronate-liposomes-control-liposomes-pbs/
Insulin	Roche	https://www.sigmaaldrich.com/NL/en/product/roche/11376497001
D-glucose	Sigma-Aldrich	https://www.sigmaaldrich.com/NL/en/product/sigma/g8270

In Vivo: Atherosclerosis initiation study

Study setup:

Groups	Sex	Age	Number (prior to experiment)	Number (after termination)	Littermates (Yes/No)	Other description
Group 1 (Control)	Female	78 ± 6 days	15	15	No	Treated with intraperitoneal vehicle injections (4% DMSO in PBS) 3 times weekly for 6 weeks while on a WTD.
Group 2 (ONX-0914)	Female	78 ± 7 days	15	15	No	Treated with intraperitoneal ONX-0914 (10 mg/kg), 3 times weekly for 6 weeks while on a WTD.

Sample Size:

15 animals per group. Atherosclerotic lesion size is the most important parameter with the largest variability. A group size of 12-15 mice is usually adequate to observe a significant difference between groups when lesion averages differ more than 25% between groups.

Inclusion Criteria / Exclusion Criteria / Randomization

Healthy female LDLr$^{-/-}$ mice were selected and randomized based on age and weight (Control; 22.1 ± 0.7 g, ONX-0914; 22.1 ± 1.15 g).

Blinding

Histological analysis was performed on blinded samples.

In Vivo: Effect of ONX-0914 on pre-existing adipose tissue

Study setup:

Groups	Sex	Age	Number (prior to experiment)	Number (after termination)	Littermates (Yes/No)	Other description
Group 1 (Baseline)	Male	73 ± 8 days	8	8	No	4 Weeks on WTD, then sacrificed for baseline of the two other groups.
Group 2 (Control)	Male	73 ± 6 days	8	8	No	11 weeks on WTD, after 4 weeks this group received intraperitoneal vehicle injections (4% DMSO in PBS) 3 times weekly.
Group 3 (ONX-0914)	Male	72 ± 9 days	8	8	No	11 weeks on WTD, after 4 weeks this group received ONX-0914 injections (4% DMSO in PBS) 3 times weekly.

Sample Size:

8 animals per group. Changes in metabolic parameters as the main interest of this experiment. Less variability expected than usually observed in atherosclerotic lesion size, therefore 8 mice per group.

Inclusion Criteria / Exclusion Criteria / Randomization

Healthy male LDLr$^{-/-}$ mice were selected, and randomized based on age and weight after 4 weeks on WTD. Baseline group weight 29.1 ± 3.5 g, Control group weight 28.59 ± 2.0 g, ONX-0914 group weight 29.1 ± 1.6 g.

Blinding

No blinding was performed.

In Vivo: Short term effect of ONX-0914 on adipose tissue and preadipocytes

Study setup:

Groups	Sex	Age	Number (prior to experiment)	Number (after termination)	Littermates (Yes/No)	Other description
Group 1 (Control)	Male	101± 5 days	6	6	No	5 weeks on a WTD prior to treatment. Then received 4 intraperitoneal control injections (4% DMSO in PBS) in 8 days.
Group 2 (1 day)	Male	92 ± 5 days	6	6	No	5 weeks on a WTD prior to treatment. Then 3 control injections (4% DMSO in PBS), and an ONX-0914 injection (10 mg/kg) a day prior to sacrifice.
Group 3 (1 week)	Male	102 ± 4 days	6	6	No	5 weeks on a WTD prior to treatment. 4control injections (4% DMSO in PBS), and an ONX-0914 injection (10 mg/kg) a day prior to sacrifice.

Sample Size

6 animals per group. Changes in immune infiltrate as the main interest of this experiment. Less variability expected than observed in atherosclerotic lesion size.

Inclusion Criteria / Exclusion Criteria / Randomization

Healthy male LDLr$^{-/-}$ mice were selected, and randomized based on age and weight after 5 weeks on WTD. Control group weight 30.2± 2.7 g, 1 day treated group weight 31.5 ± 0.5 g, 1 week treated group weight 30.4 ± 1.9 g.

Blinding

No blinding was performed.

In Vivo: Metabolic cage study

Study design:

Groups	Sex	Age	Number (prior to experiment)	Number (after termination)	Littermates (Yes/No)	Other description
Group 1 (Control)	Female	85 ± 5 days	8	8	Yes	3 weeks on a WTD prior to randomization and treatment. Then treated with (4% DMSO in PBS) 3 times weekly for 2.5 weeks.
Group 2 (ONX-0914)	Female	86 ± 11 days	8	8	Yes	3 on a WTD prior to randomization and treatment. Then ONX-0914 treatment (10 mg/kg) 3 times weekly for 2.5 weeks.

Sample Size

8 animals per group. Changes in metabolic parameters as the main interest of the experiment. A group size of 8 is sufficient to observe relevant changes in metabolism.

Inclusion Criteria / Exclusion Criteria / Randomization

Female ApoE*3-Leiden CETP mice with TC levels in serum below 8 mM after 2.5 weeks of WTD feeding were excluded. Healthy female ApoE3-Leiden CETP mice were selected, and randomized based on age, weight, fat mass and lean mass (echo MRI), TG (mM), and TC (mM) levels in serum. Group averages at randomization are listed in the table below.

Group	Age (days)	Weight (g)	Fat (g)	Lean (g)	TG (mM)	TC (mM)
Control	85 ± 7	22.1 ± 2.5	3.1 ± 1.3	18.3 ± 1.3	6.6 ± 2.0	11.1 ± 1.8
ONX-0914	86 ± 11	21.9 ± 1.6	2.9 ± 1.0	18.4 ± 0.8	6.7 ± 2.1	11.0 ± 2.1

Blinding

No blinding was performed.

In Vivo: Oral lipid tolerance test and advanced atherosclerosis

Study setup:

Groups	Sex	Age	Number (prior to experiment)	Number (after termination)	Littermates (Yes/No)	Other description
Group 1 (Control)	Female	75 ± 9 days	7	6	No	Fed a WTD for 27 weeks. Thereafter, intraperitoneal empty liposome administration at day 0, 2, 5,7. Intraperitoneal vehicle control (4% DMSO in PBS) administration at day 1, 3, 6, and 8. Fasted overnight after day 6, after which an oral lipid tolerance was performed. Sacrificed at day 9.
Group 2 (Clodronate liposomes)	Female	84 ± 9 days	7	6	No	Fed a WTD for 27 weeks. Thereafter, intraperitoneal clodronate liposome administration at day 0, 2, 5,7. Intraperitoneal vehicle control (4% DMSO in PBS) administration at day 1, 3, 6, and 8. Fasted overnight after day 6, after which an oral lipid tolerance was performed. Sacrificed at day 9.
Group 3 (ONX-0914)	Female	87 ± 11 days	7	7	No	Fed a WTD for 27 weeks. Thereafter, intraperitoneal empty liposome administration at day 0, 2, 5,7. Intraperitoneal ONX-0914 (10mg/kg) administration at day 1, 3, 6, and 8. Fasted overnight after day 6, after which an oral lipid tolerance was performed. Sacrificed at day 9.
Group 4 (clodronate liposomes and ONX-0914)	Female	83 ± 10	7	7	No	Fed a WTD for 27 weeks. Thereafter, intraperitoneal clodronate liposome administration at day 0, 2, 5,7. Intraperitoneal ONX-0914 (10 mg/kg) administration at day 1, 3, 6, and 8. Fasted overnight after day 6, after which an oral lipid tolerance was performed. Sacrificed at day 9.

Sample Size

7 female LDLr$^{-/-}$ animals per group. Changes in oral lipid uptake and general inflammation as major study parameters. Less variability expected than observed in atherosclerotic lesion size.

Inclusion Criteria / Exclusion Criteria / Randomization

Healthy female LDLr$^{-/-}$ mice. Randomization based on age and weight after 27 weeks on a WTD.

Blinding

No blinding was performed.

In Vivo: Gastric Emptying

Study setup:

Groups	Sex	Age	Number (prior to experiment)	Number (after termination)	Littermates (Yes/No)	Other description
Group 1 (Control)	Male	140 ± 0 days	3	3	Yes	Fed a WTD for 3 weeks. Control treated (4% DMSO in PBS intraperitoneally) for a week. Thereafter, oral administration of GastroSense 750 and cervical dislocation 30 minutes later.
Group 2 (ONX-0914)	Male	140 ± 0 days	3	3	Yes	Fed a WTD for 3 weeks. ONX-0914 treated (3 times weekly, intraperitoneally) for a week. Thereafter, oral administration of GastroSense 750 and cervical dislocation 30 minutes later.

Sample Size

3 male LDLr$^{-/-}$ mice per group. Changes in gastric emptying (marked by GastroSence 750 signal) as study parameter. Setup as a small pilot experiment, the difference in GastroSense signal in the stomach between treatment groups was already significant with this sample size.

Inclusion Criteria / Exclusion Criteria / Randomization

Mice were all from the same litter, and were randomized on weight prior to WTD feeding (control; 28.7 ± 0.9 g, ONX-0914; 28.7 ± 0.5 g)

Blinding

No blinding was performed.

In Vivo: Advanced atherosclerosis in aged LDLr$^{-/-}$ mice

Study setup:

Groups	Sex	Age	Number (prior to experiment)	Number (after termination)	Littermates (Yes/No)	Other description
Group 1 (Control)	Male	469,6 ± 8,4 days	14	14	Yes	
Group 2 (ONX-0914)	Male	470 ± 10,4 days	13	12	Yes	

Sample Size

Changes in immune phenotype as main readout parameter. Because a higher variability in immune parameters and some dropouts due to the advanced age of the mice were anticipated, group sizes that are a little bit larger than we would typically use for the immunophenotyping of immunomodulatory interventions were used.

Inclusion Criteria / Exclusion Criteria / Randomization

Healthy male LDLr$^{-/-}$ mice were randomized based on age (Control: 469.6 ± 8.4 days, ONX-0914: 470 ± 10,4 days), weight (Control: 37.8 ± 4.8 g, ONX-0914: 38.9 ± 5.2g), and plasma TC levels (298.3 ± 90.2 mg/dL, ONX-0914: 307.8 ± 61.0 mg/dL).

Blinding

Histological analysis was performed on blinded samples.

In Vivo: Atherosclerosis regression

Study setup:

Groups	Sex	Age	Number (prior to experiment)	Number (after termination)	Littermates (Yes/No)	Other description
Group 1 (baseline)	Female	137 ± 18 days	12	12	No	Mice were fed a WTD for 6 weeks after which they were sacrificed.
Group 2 (Control)	Female	137 ± 11 days	12	12	Yes	Mice were fed a WTD for 6 weeks after which they were switched to a chow diet for 6 weeks, while receiving vehicle (4% DMSO in PBS) intraperitoneally 3 times weekly, and then sacrificed.
Group 3 (ONX-0914)	Female	138 ± 11 days	12	12	Yes	Mice were fed a WTD for 6 weeks after which they were switched to a chow diet for 6 weeks, while receiving ONX-0914 (10 mg/kg) intraperitoneally 3 times weekly, and then sacrificed.

Sample Size

12 female LDLr$^{-/-}$ were used to be able to assess changes in atherosclerotic lesion size upon treatment with ONX-0914. Upon switching the mice back to a chow diet, the Th1 levels rapidly declined in the control and ONX-0914 treated group, compared to the baseline group (data not shown). Atherosclerotic lesions appeared very collagen rich and stable in the control and ONX-0914 group (data not shown). These observations indicate that in LDLr$^{-/-}$ mice, returning cholesterol levels back to baseline levels is sufficient to also reduce inflammation, while in patients often a residual inflammatory risk remains.

Inclusion Criteria / Exclusion Criteria / Randomization

Control and ONX-0914 groups were randomized based on age, weight (Control: 29.0 ± 3.4 g, ONX-0914; 27.3 ± 3.7g), plasma cholesterol levels (Control; 1504.2 ± 209.9 mg/dL, ONX-0914; 1465.9 ± 264.3 mg/dl), and plasma TG levels (Control; 506.4 ± 105.9 mg/dL, ONX-0914; 461.7 147.8 mg/dL). The baseline group was age matched with the other 2 groups.

Blinding

Histological analysis was performed on blinded samples.

In Vivo: Glucose tolerance test

Study setup:

Groups	Sex	Age	Number (prior to experiment)	Number (after termination)	Littermates (Yes/No)	Other description
Group 1 (Control)	Male	62 ± 0 days	7	6	Yes	Mice were fed a WTD for 6 weeks after which they were control treated 3 times weekly with 4% DMSO in PBS intraperitoneally for 3 weeks.
Group 2 (ONX-0914)	Male	62 ± 0 days	7	7	Yes	Mice were fed a WTD for 6 weeks after which they were treated 3 times weekly with ONX-0914 in PBS intraperitoneally for 3 weeks.

Sample Size

7 male LDLr$^{-/-}$ were used per group to assess the glucose sensitivity.

Inclusion Criteria / Exclusion Criteria / Randomization

Control and ONX-0914 groups were randomized based on age (62 ± 0 days for both groups), body weight prior to WTD feeding (Control: 25.6 ± 2.0 g, ONX-0914; 25.8 ± 1.6 g), cholesterol levels prior to WTD feeding (Control; 171.66 ± 39.8 mg/dL, ONX-0914; 178.81 ± 60.58 mg/dl), and weight after 5 weeks of WTD (Control: 30.6 ± 2.4 g, ONX-0914; 30.7 ± 2.2 g).

Blinding

Blood glucose measurements were blinded.

In Vivo: Insulin tolerance test

Study setup:

Groups	Sex	Age	Number (prior to experiment)	Number (after termination)	Littermates (Yes/No)	Other description
Group 1 (Control)	Male	77 ± 9 days	7	6	Yes	Mice were fed a WTD for 6 weeks after which they were control treated 3 times weekly with 4% DMSO in PBS intraperitoneally for 3 weeks.
Group 2 (ONX-0914)	Male	76 ± 10 days	7	7	Yes	Mice were fed a WTD for 6 weeks after which they were treated 3 times weekly with ONX-0914 in PBS intraperitoneally for 3 weeks.

Sample Size

7 male LDLr$^{-/-}$ were used per group to assess the insulin sensitivity.

Inclusion Criteria / Exclusion Criteria / Randomization

Control and ONX-0914 groups were randomized based on age (Control: 77 ± 9 days, ONX-0914; 76 ± 10 days), body weight prior to WTD feeding (Control: 27.8 ± 1.4 g, ONX-0914; 27.5 ± 2.2 g), cholesterol levels prior to WTD feeding (Control; 164.52 ± 30.38 mg/dL, ONX-0914; 166.9 ± 31.33 mg/dl), and weight after 5 weeks of WTD (Control: 32.0 ± 2.2 g, ONX-0914; 32.0 ± 3.1 g).

Blinding

Blood glucose measurements were blinded.
